# Interleukin 9 mediates T follicular helper cell activation to promote antibody responses

**DOI:** 10.3389/fimmu.2024.1441407

**Published:** 2024-09-30

**Authors:** Taiki Sato, Ippei Ikegami, Masahiro Yanagi, Takeshi Ohyu, Taiki Sugaya, Shotaro Shirato, Masanobu Tanemoto, Shiori Kamiya, Kohei Kikuchi, Yuka Kamada, Takehito Nakata, Ryuta Kamekura, Akinori Sato, Ken-ichi Takano, Masahiro Miyajima, Atsushi Watanabe, Shingo Ichimiya

**Affiliations:** ^1^ Department of Human Immunology, Research Institute for Immunology, Sapporo Medical University School of Medicine, Sapporo, Japan; ^2^ Department of Thoracic Surgery, Sapporo Medical University School of Medicine, Sapporo, Japan; ^3^ Department of Otolaryngology and Head and Neck Surgery, Sapporo Medical University School of Medicine, Sapporo, Japan; ^4^ Department of Physical Therapy, Faculty of Human Sciences, Hokkaido Bunkyo University, Eniwa, Japan

**Keywords:** follicular mantle zone B cells, germinal center response, IL-9, ILC2, leukotrienes, T follicular helper cells

## Abstract

Antigen-specific humoral responses are orchestrated through complex interactions among immune cells in lymphoid tissues, including the collaboration between B cells and T follicular helper (Tfh) cells. Accumulating evidence indicates a crucial role for interleukin-9 (IL-9) in the formation of germinal centers (GCs), enhancing the generation of class-switched high-affinity antibodies. However, the exact function of IL-9 in Tfh cell regulation remains unclear. In this study, we examined the humoral immune responses of CD4^Cre/+^Il9ra^fl/fl^ mice, which lack an IL-9-specific receptor in Tfh cells. Upon intraperitoneal immunization with sheep red blood cells (SRBCs), CD4^Cre/+^Il9ra^fl/fl^ mice displayed diminished levels of SRBC-specific IgG antibodies in their sera, along with reduced levels of GC B cells and plasma cells. Notably, Il9ra-deficient Tfh cells in the spleen exhibited decreased expression of their signature molecules such as B-cell lymphoma 6, C-X-C chemokine receptor 5, IL-4, and IL-21 compared to control mice. In models of allergic asthma induced by house dust mite (HDM) inhalation, CD4^Cre/+^Il9ra^fl/fl^ mice failed to elevate serum levels of HDM-specific IgE and IgG. This was accompanied by reductions in Tfh cells, GC B cells, and plasma cells in mediastinal lymph nodes. Furthermore, group 2 innate lymphoid cells (ILC2s) were identified as producers of IL-9 under immunizing conditions, possibly induced by leukotrienes released by activated IgD^+^ B cells around the T-B border. These observations may indicate the critical role of IL-9 receptor signaling in the activation of Tfh cells, with ILC2s potentially capable of supplying IL-9 in organized lymphoid tissues.

## Introduction

Humoral immunity is a fundamental component of the host defense system, offering an efficient mechanism of protection against diverse foreign antigens and pathogens (1, 2). T follicular helper (Tfh) cells, a specialized subset of helper CD4^+^ T cells defined by the lineage-specifying transcription factor B-cell lymphoma 6 (Bcl6), play a crucial role in the development of germinal center (GC) B cells, ensuring the establishment of high-affinity and long-lived humoral immunity. Dysfunction of Tfh activity is implicated in various conditions characterized by aberrant antibody production, highlighting the significance of Tfh cells in infection, allergy, and autoimmunity (3). Therefore, there is a significant interest in investigating the regulatory mechanisms governing Tfh cells to better understand the mechanisms of specific humoral immune responses, enhance vaccine efficacy, and address disease pathogenesis.

To achieve effective humoral responses, cell surface molecules expressed on Tfh cells regulate their activation and distribution, facilitating contact-dependent interaction with B cells. These molecules include C-X-C chemokine receptor 5 (CXCR5), programmed cell death protein 1 (PD-1), sphingosine 1-phosphate receptor (S1PR), inducible T cell co-stimulator (ICOS), CD40 ligand (CD40L), and ephrin type-B receptor (4–8). To instruct B cell differentiation, Tfh cells produce common γ-chain (γc) family cytokines such as interleukin-4 (IL-4) and IL-21, which specifically promote and maintain GC B cells in the dark and light zones, resulting in the robust generation of specific antibody-secreting cells and memory B cells (9–11). In addition to these cytokines, it has recently been suggested that a population of Tfh cells can produce IL-9 of the γc family, which binds to a heterodimeric receptor consisting of the γc and cytokine-specific IL-9 receptor α-chain (12, 13). While IL-9 is recognized to exert multiple functions in various types of cells, it has been noted that IL-9 plays a role in T-cell biology, particularly in pathologic conditions including allergy and autoimmunity affecting specific tissue lesions (14–16). IL-9 also regulates GC B cells to optimize the memory B cell compartment and potentiates B cell responses by enhancing the production of baseline and antigen-specific antibodies, such as IgG and IgE (17–19). In general, the IL-9 receptor allows the activation of intracellular signaling cascades including Janus kinase-signal transducer and activator of transcription (JAK-STAT), phosphatidylinositol-3 (PI-3) kinase, and mitogen-activated protein (MAP) kinase pathways (13, 20). IL-9 receptor (Il9r)-deficient mice have impaired specific antibody responses (19); however, the functional significance of IL-9 in modulating Tfh cells to promote specific humoral immune responses is incompletely understood.

To study the role of IL-9 in regulating Tfh cell function, we generated and analyzed CD4-specific Il9r-deficient mice, which carry floxed alleles (Il9ra^fl/fl^) for the entire *Il9ra* gene encoding the IL-9 receptor α-chain (21). Immunization models using sheep red blood cells (SRBCs) in CD4-specific Il9ra-deficient mice revealed decreased production of SRBC-specific antibodies and ineffective GC formation in their secondary lymphoid tissues. This deficiency likely stemmed from the defective function of Il9ra-deficient Tfh cells, which exhibited low expression of Bcl6, CXCR5, and cytokines IL-4 and IL-21 (22). In our experiments, group 2 innate lymphoid cells (ILC2s) were considered as the primary producers of IL-9 in secondary lymphoid tissues (23, 24). Additional results suggest a potential mechanism for activating Tfh cell function in collaboration with ILC2s and activated IgD^+^ B cells, which can produce arachidonate 5-lipoxygenase (Alox5)-mediated leukotrienes for ILC2s (25–27). Further discussion on the pathological significance of IL-9 on Tfh cells during chronic inflammation was expanded based on results from the study of allergic asthma models induced by house dust mite (HDM) inhalation.

## Materials and methods

### Clinical specimens

Tonsil samples for the analysis of lymphocytes were collected from patients undergoing tonsillectomy at Sapporo Medical University Hospital due to tonsillar hypertrophy or recurrent tonsillitis. The protocol for collecting and analyzing surgical specimens, conducted in accordance with ethical standards relevant to human research under the Declaration of Helsinki, was approved by the Institutional Review Board of Sapporo Medical University Hospital (IRB #25-39). All participants provided written informed consent before the study.

### Mice and immunization

CD4^Cre/+^Il9ra^fl/fl^ and FoxP3^GFP/DTR^CD4^Cre/+^Il9ra^fl/fl^ mice were generated from Il9ra^fl/+^ mice with a C57BL/6 background (CDB0034E, RIKEN Center for Biosystems Dynamics Research, Kobe, Japan). Il9ra^fl/+^ mice were designed to enable specific ablation of the entire *Il9ra* gene using CRISPR/Cas9 genome editing technology (21). CD4^Cre/+^ (B6.Cg-Tg(Cd4-cre)1Cwi/BfluJ), FoxP3^GFP/DTR^ (B6.129(Cg)-Foxp3^tm3(DTR/GFP)Ayr^/J), and MD4 (C57BL/6-Tg(IghelMD4)4Ccg/J) mice were obtained from the Jackson Laboratory (Bar Harbor, ME), and wild-type C57BL/6 mice were purchased from Sankyo Laboratory (Tokyo, Japan). The mice were bred and housed under specific pathogen-free conditions in the animal facility at Sapporo Medical University. Age-matched (6-12 weeks) and sex-matched littermates in each group were used for the analysis. For immunization, mice were intraperitoneally administered 200 μL of 20% SRBCs (Japan Bio Serum, Tokyo, Japan) or 100 μg of hen egg lysozyme (HEL; Roche, Basel, Switzerland) in conjunction with CFA (Fujifilm Wako Pure Chemical, Tokyo, Japan). Sera were collected from tail veins and stored at −80°C until examinations. All animal experiments were conducted following institutional and national animal welfare guidelines, following the approved protocol (#17-120, #23-043) of Sapporo Medical University for the care and use of animals.

### Antibodies, flow cytometry, and cell sorting

Lymphocytes in single-cell suspensions were prepared from tissues by carefully and gently mashing the tissues, followed by density-gradient centrifugation with Lympholyte (Cedarlane, Burlington, Canada). To analyze the mouse cells by flow cytometry or cell sorting, the cells were pretreated with an anti-mouse CD16/CD32 mAb (clone 2.4G2; BD Biosciences) and stained using a standard protocol. Fluorochrome-conjugated mAbs for cell staining are summarized in **Supplementary Table S1**. ILCs were defined as a CD45^+^CD127^+^ population negative for lineage markers, including B220, CD3, CD11b, CD11c, Gr-1, NK1.1, TCRβ, TCRγδ, and TER119. The cells were analyzed using FACS Canto I (BD Biosciences) or subjected to sorting with FACS Aria II or III (BD Biosciences), in combination with magnetic bead sorting (Thermo Fisher Scientific, Waltham, MA). The purity of sorted cells reached over 95% after validation by reanalysis using FACS Canto I. In each experiment, cells were first gated on lymphocytes based on forward and side scatter, then on single cells after doublet discrimination, and on live cells using 7-AAD before gating on the cell subsets of interest. For intracellular protein staining such as transcription factors and cytokines, the cells were analyzed using a standard protocol as described previously (28). All data were analyzed using FACS DiVA and FlowJo software (BD Biosciences).

### RT-qPCR analysis

Total RNA was extracted from cells with TRIzol RNA isolation reagent (Thermo Fisher Scientific). Single-strand cDNA was synthesized from the total RNA using a high-capacity cDNA reverse transcription kit (Thermo Fisher Scientific). Quantitative PCR analysis was conducted to detect gene-specific products using TaqMan probes with Light Cycler 96 System (Roche). The TaqMan probes were provided by Thermo Fisher Scientific: *Il9ra* (FAM-MGB, Mm00434313_m1) and *Gapdh* (VIC-MGB, Mm99999915_g1) as an endogenous control.

### Cell culture experiments of T cells and ILC2s

To study the functional effects of IL-9 on CD4^+^ T cell subsets, 2.0 × 10^5^ cells were cultured in 200 μl of RPMI-1640 medium (Fujifilm Wako Pure Chemical) containing 10% heat-inactivated FCS, 100 units/mL penicillin, 50 μg/mL streptomycin, 55 μM 2-mercaptoethanol, 10 mM Hepes (pH 7.4), and 1.5 μg/mL anti-CD28 mAb (clone 37.51; BioLegend, San Diego, CA) supplemented with or without 20 ng/mL recombinant mouse IL-9 (Peprotech, Cranbury, NJ) in a 96-well plate coated with 10 μg/mL anti-CD3 mAb (clone 145-2C11; BD Biosciences). The cells were subsequently incubated at 37°C in a humidified atmosphere of 5% CO_2_ for 3 days. Then, the cells were stained with cell surface markers to detect Tfh cells and subjected to intracellular flow cytometry after staining for Bcl6. ILC2s were isolated from the spleen of SRBC-immunized mice. After 7 days of immunization, single-cell suspensions were prepared using density-gradient centrifugation with Lympholyte (Cedarlane). CD19^+^ B cells and CD3^+^ T cells were removed using the MagniSort mouse CD19 and CD3 positive selection kits (Thermo Fisher Scientific). The remaining cells were treated using a mouse ILC2 enrichment kit (Stemcell Technologies, Vancouver, Canada). A total of 5.0 × 10^4^ ILC2s were incubated in RPMI-1640 medium containing 100 units/mL penicillin and 50 μg/mL streptomycin, supplemented with 1 μM leukotrienes (Cayman Chemical Company, Ann Arbor, MI) and 1:1500 GolgiStop (BD Biosciences). The cells were then incubated at 37°C in a humidified atmosphere of 5% CO_2_ for 6 h and subjected to intracellular flow cytometry to measure IL-9.

### Laser confocal microscopy

Spleen tissues frozen in Tissue-Tek OCT compound were cryosectioned into 15-μm tissue sections and fixed in ice-cold acetone. For immunostaining, tissue sections were incubated with primary antibodies in a moisture chamber at 4°C overnight, followed by staining with fluorochrome-conjugated secondary antibodies (**Supplementary Table S1**). The tissue sections were stained with Alexa Fluor 594-conjugated lectin PNA (Invitrogen, Waltham, MA) to detect GCs and mounted with DAPI (Invitrogen) to visualize the nuclei. Subsequently, the tissue sections were analyzed using ELYRAS.1 LSM780 laser confocal microscope (Carl Zeiss, Jena, Germany), and the acquired images were analyzed using ZEN image examiner software (Carl Zeiss).

### HDM-induced bronchial asthma model

To induce allergic airway inflammation with HDM, the mice were anesthetized with isoflurane, and 50 μL of PBS containing 25 μg of HDM extract (Greer Laboratories, Lenoir, NC) was administered intranasally three times per week for 5 consecutive weeks. Control mice received 50 μL of PBS instead of the HDM solution. At the end of the experiments, mice were euthanized, and serum samples and mediastinal lymph nodes were collected for further analysis. Lung tissues were fixed in 10% buffered formalin solution, embedded in paraffin, and prepared as FFPE tissue sections. These sections were stained with hematoxylin and eosin (HE) or Periodic Acid-Schiff (PAS) for examination under a light microscope.

### ELISA

A 96-well microtiter plate coated with protein extracts from SRBCs in bicarbonate buffer was used to determine anti-SRBC Ig titers in sera. Ig isotypes were determined using isotype-specific ELISA (Southern Biotech, Birmingham, AL). HDM-specific IgG was detected using a high-binding plate coated with 5 μg/mL HDM (Greer Laboratories). HDM-specific IgE was analyzed using an ELISA kit for anti-HDM IgE antibodies according to the manufacturer’s protocols (Chondrex, Woodinville, WA). LTB4 and LTC4 in the culture supernatants were analyzed using ELISA kits for LTB4 and LTC4, respectively, according to the manufacturer’s instructions (Cayman Chemical Company). The OD value was measured as the absorbance using a microplate reader (Multiskan FC, Thermo Fisher Scientific).

### Statistical analysis

All statistical tests were performed using GraphPad Prism (version 7.0; GraphPad Software, La Jolla, CA). The unpaired *t-*test was used to analyze the differences between the two groups. One-way ANOVA with Tukey’s multiple comparison test was used to compare multiple groups. *p-*values below 0.05 were considered statistically significant differences between groups and are denoted by asterisks on graphs: *p < 0.05, **p < 0.01, ***p < 0.001, ****p < 0.0001. When the test results show no significant difference, this is not specifically noted in the graph.

## Results

### Tfh cells express IL-9 receptor

**Figure 1:**
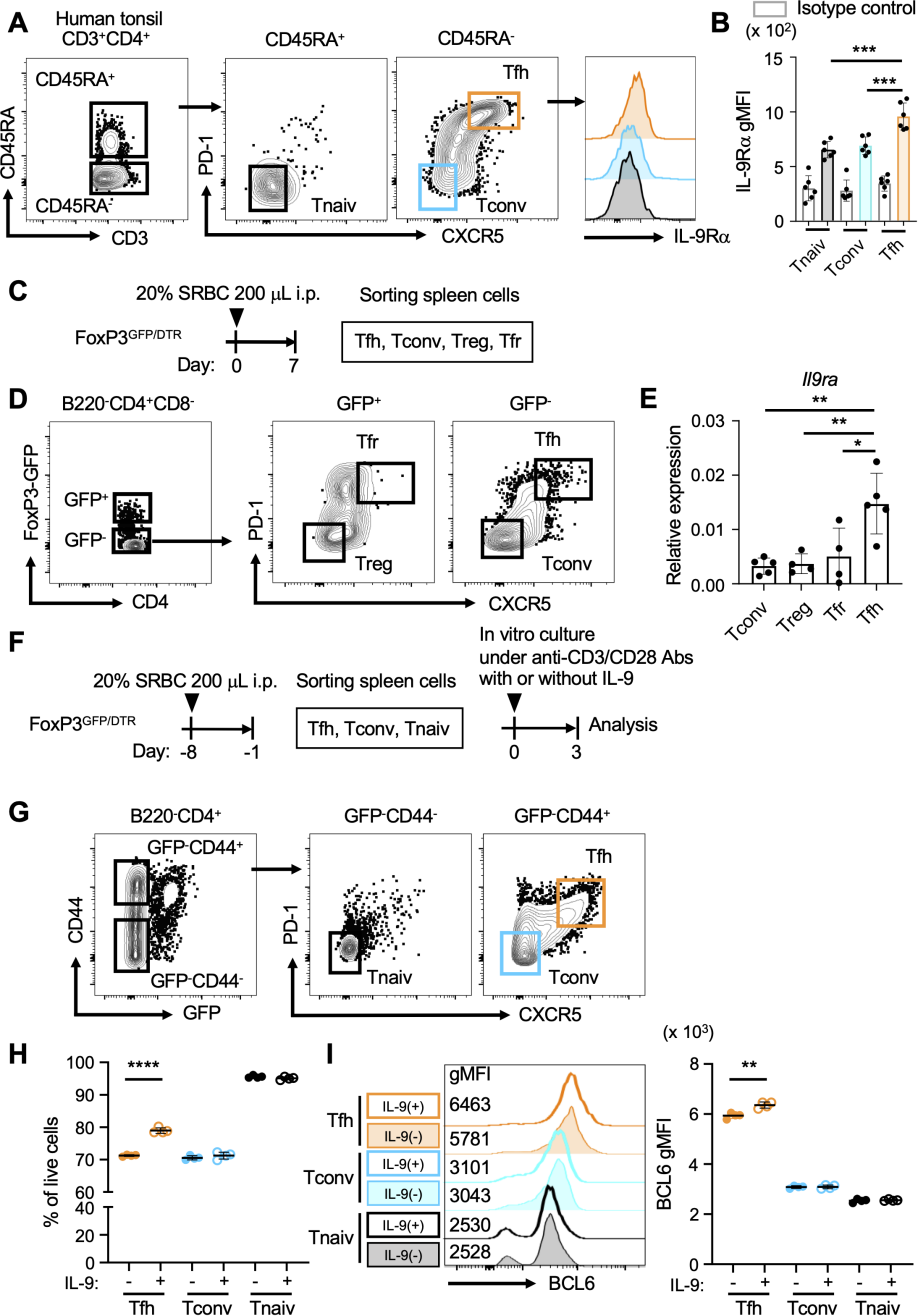
Tfh cells express IL-9 receptor. **(A)** Gating strategy for CD4^+^ T cell populations (CD3^+^CD4^+^) derived from human tonsillar lymphocytes, including Tfh cells (CD45RA^-^CXCR5^+^PD-1^+^), naive CD4^+^ T cells (Tnaiv cells; CD45RA^+^CXCR5^-^PD-1^-^), and conventional CD4^+^ T cells (Tconv cells; CD45RA^-^CXCR5^-^PD-1^-^). Histograms indicate the surface expression of IL-9RA on these CD4^+^ T cell populations. **(B)** Expression levels of IL-9RA on Tfh cells, Tnaiv cells, and Tconv cells in A using the geometric mean fluorescence intensity index (gMFI). ***p = 0.0002 (Tnaiv cells vs. Tfh cells), ***p = 0.0006 (Tconv cells vs. Tfh cells). Each dot represents an individual result from a tonsil, with a total of six tonsils analyzed. Data represent the mean ± SD. **(C)** Experimental protocol for the immunization of FoxP3^GFP/DTR^ mice. SRBCs were administered intraperitoneally, followed by the isolation and sorting of CD4^+^ T cell populations from splenocytes. **(D)** Sorting strategy for CD4^+^ T cell populations (B220^-^CD4^+^CD8^-^) from the splenocytes of FoxP3^GFP/DTR^ mice in C, including Tfh cells (GFP^-^CXCR5^+^PD-1^+^), Tfr cells (GFP^+^CXCR5^+^PD-1^+^), Treg cells (GFP^+^CXCR5^-^PD-1^-^), and Tconv cells (GFP^-^CXCR5^-^PD-1^-^). **(E)** Expression levels of *Il9ra* transcripts in CD4^+^ T cell subsets as measured using RT-qPCR. Each dot represents the average value of a group of mice (n = 8-10) in a single experiment. Data from 4-5 experiments are presented as the mean ± SD. **p = 0.0024 (Tconv cells vs. Tfh cells), **p = 0.0051 (Treg cells vs. Tfh cells), *p = 0.0135 (Tfr cells vs. Tfh cells). Similar results were obtained across three independent experiments. **(F)** Experimental protocol for *in vitro* culture of CD4^+^ T cell populations from FoxP3^GFP/DTR^ mice. SRBCs were administered intraperitoneally, and CD4^+^ T cell populations were isolated from splenocytes. Subsequently, CD4^+^ T cell populations were cultured with anti-CD3/CD28 mAbs in the presence or absence of IL-9. **(G)** Sorting strategy for CD4^+^ T cell populations (B220^-^CD4^+^) from the splenocytes of FoxP3^GFP/DTR^ mice in F, including Tfh cells (GFP^-^CD44^+^CXCR5^+^PD-1^+^), Tnaiv cells (GFP^-^CD44^-^CXCR5^-^PD-1^-^), and Tconv cells (GFP^-^CD44^+^CXCR5^-^PD-1^-^). **(H)** Percentages of each CD4^+^ T cell population per total live cells, as assessed using flow cytometry with 7-AAD. ****p < 0.0001 (without IL-9 vs. with IL-9 in Tfh cells). **(I)** Expression levels of Bcl6 in each CD4^+^ T cell population, as analyzed using intracellular flow cytometry with histgrams and gMFI values. **p = 0.0024 (without vs. with IL-9 in Tfh cells). Each dot represents the average of triplicates in one experiment, and the data indicate the mean value. Similar results were obtained in two independent experiments. Statistical significance was analyzed using Tukey’s multiple comparisons test **(B, E)** and the unpaired *t*-test **(H, I)**.

First, we examined the expression profile of the IL-9 receptor α-chain (IL-9RA, CD129) on CD4^+^ T cell populations in human tonsil lymphocytes through flow cytometry. The results indicated that IL-9RA was present on Tfh cells (CD3^+^CD4^+^CD45RA^-^CXCR5^+^PD-1^+^; **Figure 1A**). The expression levels of IL-9RA on Tfh cells surpassed those on naive CD4^+^ T cells (Tnaiv cells; CD3^+^CD4^+^CD45RA^+^CXCR5^-^PD-1^-^) and conventional CD4^+^ T cells (Tconv cells; CD3^+^CD4^+^CD45RA^-^CXCR5^-^PD-1^-^), suggesting that Tfh cells are likely more sensitive to IL-9 compared to Tnaiv and Tconv cells (**Figure 1B**). Since T follicular regulatory (Tfr) cells present both CXCR5 and PD-1 like Tfh cells, we next investigated CD4^+^ T cell populations in FoxP3^GFP/DTR^ mice. In this model, Tfr cells and Tfh cells can be separately analyzed based on the expression levels of FoxP3, indicated by GFP expression (29). FoxP3^GFP/DTR^ mice were intraperitoneally immunized with SRBCs as T-dependent antigens, and CD4^+^ T cell populations in the spleen were sorted for the analysis of *Il9ra* expression (**Figures 1C, D**). The results revealed that Tfh cells (GFP^-^CXCR5^+^PD-1^+^) exhibited higher levels of *Il9ra* transcripts compared to Tfr cells (GFP^+^CXCR5^+^PD-1^+^), regulatory T cells (Treg cells; GFP^+^CXCR5^-^PD-1^-^), and Tconv cells (GFP^-^CXCR5^-^PD-1^-^; **Figure 1E**). To further understand the functional implications of IL-9 on Tfh cells, *in vitro* culture experiments were performed using sorted CD4^+^ T cell populations from the spleen of FoxP3^GFP/DTR^ mice challenged with SRBCs (**Figures 1F, G**). The results implied that IL-9 could promote the viability of Tfh cells (GFP^-^CD44^-^CXCR5^+^PD-1^+^) and further facilitate their expression of Bcl6 (**Figures 1H, I**). In contrast, IL-9 did not seem to actively influence the viability and induction of Bcl6 expression in Tnaiv (GFP^-^CD44^-^CXCR5^-^PD-1^-^) and Tconv cells (GFP^-^CD44^+^CXCR5^-^PD-1^-^). Collectively, these observations suggest that IL-9 may play a pivotal role in regulating the functional integrity of Tfh cells.

### IL-9 receptor signaling in Tfh cells promotes GC formation

**Figure 2:**
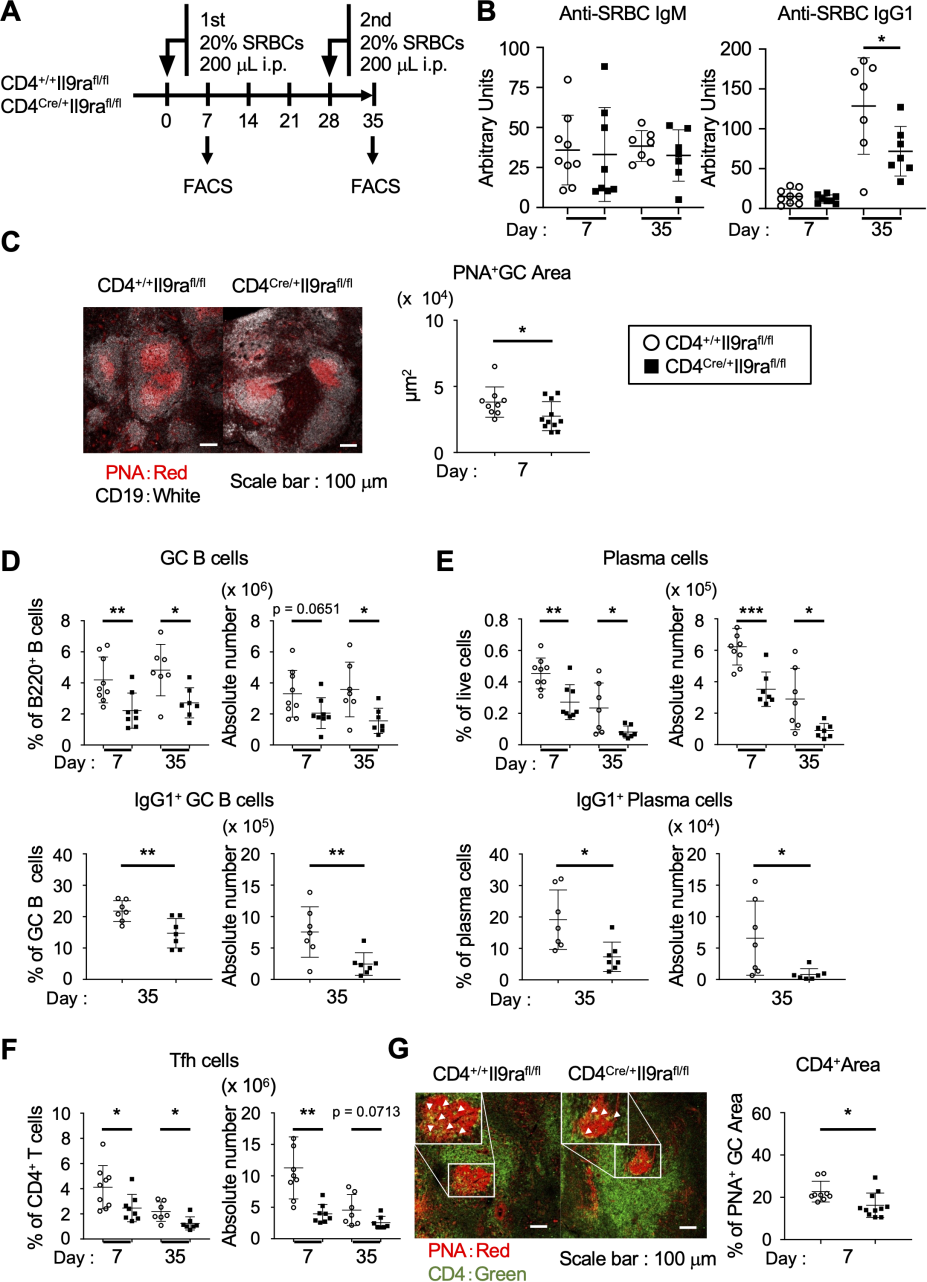
IL-9 assists Tfh cells in promoting GC formation. **(A)** Experimental protocol for the immunization of CD4^Cre/+^Il9ra^fl/fl^ and control CD4^+/+^Il9ra^fl/fl^ mice. Sera were collected from the tail vein. **(B)** Serum levels of SRBC-specific IgM and IgG. *p = 0.0476. **(C)** Representative confocal images of spleen sections, with B cells (CD19^+^) in white and GCs (PNA^+^) in red. Graphs show individual values of PNA^+^ GC areas in a tissue section, calculated using Zen software. *p = 0.0491. **(D–F)** Cell populations in the spleen (**Supplementary Figures S3A, B**). The percentages and cell numbers of each cell population are shown. **(D)** GC B cells (percentages, **p = 0.0078, *p = 0.0132; cell numbers, *p* = 0.0651, *p = 0.0173) and IgG1^+^ GC cells (percentages, **p = 0.0070; cell numbers, **p = 0.0098). **(E)** Plasma cells (percentages, **p = 0.0027, *p = 0.0293; cell numbers, ***p = 0.0003, *p = 0.0212) and IgG1^+^ plasma cells (percentages, *p = 0.0119; cell numbers, *p = 0.0251). **(F)** Tfh cells (percentages, *p = 0.0332, *p = 0.0202; cell numbers, **p = 0.0011, *p* = 0.0713). **(G)** Representative confocal images of spleen sections visualized for CD4 (green) and PNA (red). Graphs show the percentages of CD4^+^ areas localized within the PNA^+^ GC areas measured in CD4^Cre/+^Il9ra^fl/fl^ and control CD4^+/+^Il9ra^fl/fl^ mice 7 days after immunization with SRBCs. Imaging data were analyzed using ImageJ software. *p = 0.0162. Data represent the mean ± SD of 7-11 mice per group. Statistical significance was analyzed using the unpaired *t*-test **(B–G)**. Similar results were obtained in two-four independent experiments.

To study the physiological significance of IL-9 in the regulation of Tfh cells, we generated and analyzed CD4^Cre/+^Il9ra^fl/fl^ mice, which carry a floxed allele covering the entire *Il9ra* gene (21). In these mice, the *Il9ra* gene is ablated by Cre recombinase under the transcriptional control of the *Cd4* promoter element. In CD4^Cre/+^Il9ra^fl/fl^ mice, Tfh cells lost the IL-9 receptor, while the expression of IL-9 receptor was maintained in memory B cells (**Supplementary Figures S1A–C**; reference 19). In steady state, CD4^Cre/+^Il9ra^fl/fl^ mice exhibited normal T cell development in the thymus and immune cell compositions in the spleen, including T and B cells, as well as dendritic cells, compared to control CD4^+/+^Il9ra^fl/fl^ mice (**Supplementary Figures S2A–D**). In response to intraperitoneal administration of SRBCs, CD4^Cre/+^Il9ra^fl/fl^ mice failed to elevate the serum titer of anti-SRBC IgG1 antibodies to the level of control mice upon the secondary immunization (**Figures 2A, B**). Histopathologic examination using laser confocal microscopy revealed poor GC formation in the spleen of CD4^Cre/+^Il9ra^fl/fl^ mice, contrasting with that of control mice (**Figure 2C**). Accordingly, flow cytometric analysis of spleen cells revealed reduced levels of total GC B cells and plasma cells, as well as IgG1-expressing cells, in CD4^Cre/+^Il9ra^fl/fl^ mice compared to the control CD4^+/+^Il9ra^fl/fl^ mice (**Figures 2D, E**, **Supplementary Figure S3A**). Notably, Tfh cells in the spleen of CD4^Cre/+^Il9ra^fl/fl^ mice were significantly decreased compared to the control mice, even though the total number of CD4^+^ T cells was comparable (**Figure 2F**, **Supplementary Figures S3B, C**). Further studies of tissue sections of the spleen also demonstrated a reduced number of CD4^+^ T cells within GCs in CD4^Cre/+^Il9ra^fl/fl^ mice (**Figure 2G**). Taken together, the lower levels of IgG1-expressing GC B cells and plasma cells coincided with a tendency toward reduced Tfh cell levels in CD4^Cre/+^Il9ra^fl/fl^ mice. We cannot definitely exclude that *Il9ra* deficiency qualitatively impacts other CD4^+^ cells that might directly or indirectly promote GCs and humoral immunity. However, our observations rather suggest a functional deficit of CD4^Cre/+^Il9ra^fl/fl^ Tfh cells in the general formation of GCs under immunizing conditions. In this context, IL-9 receptor signaling might be necessary to control Tfh cell integrity for antigen-specific B cell responses through GC reactions.

### IL-9 receptor signaling induces the expression of Bcl6, IL-4, and IL-21 in Tfh cells

**Figure 3:**
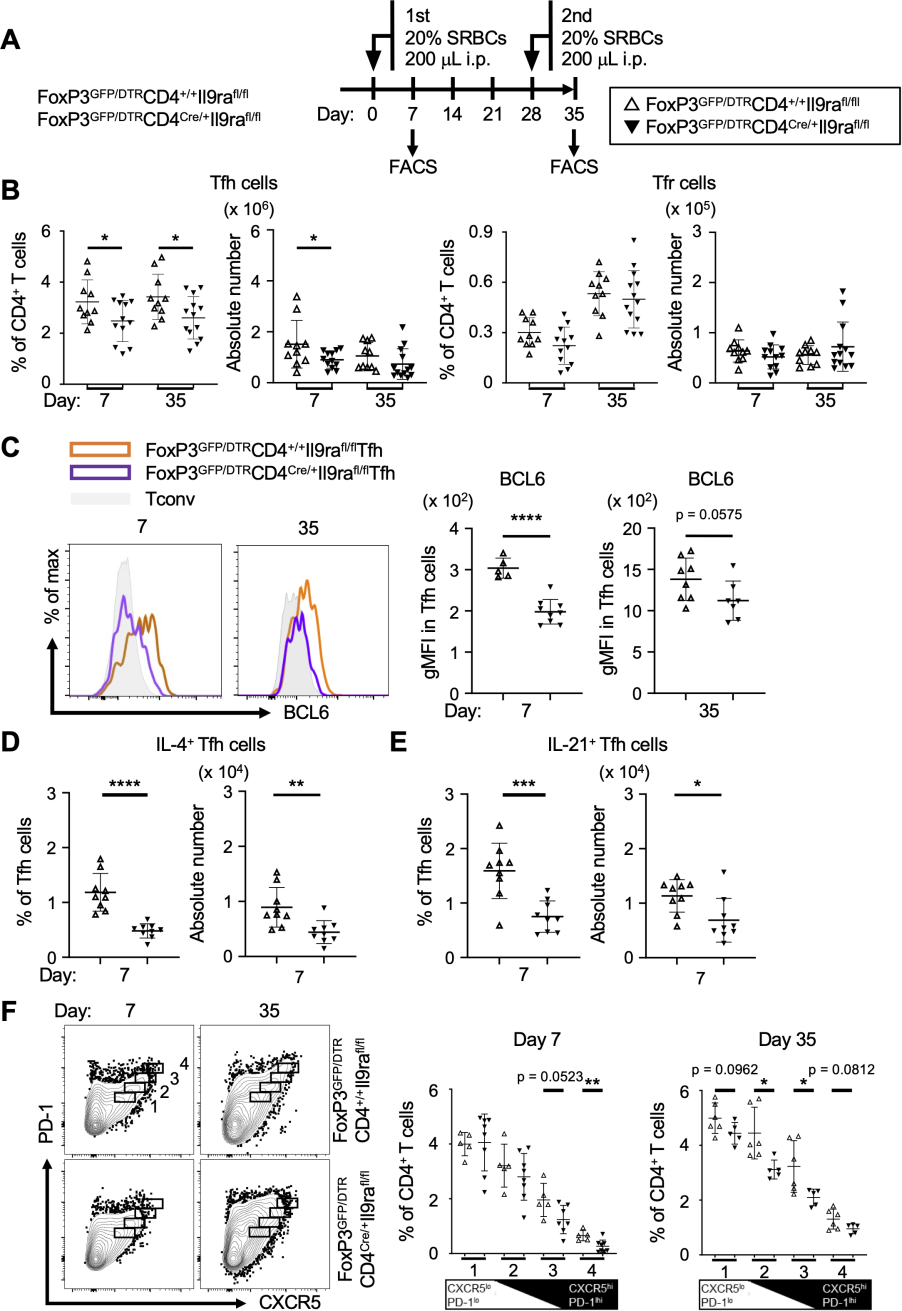
IL-9 induces Tfh cells to express Bcl6, IL-4, and IL-21. **(A)** Experimental protocol for the immunization and analysis of FoxP3^GFP/DTR^CD4^Cre/+^Il9ra^fl/fl^ and control FoxP3^GFP/DTR^CD4^+/+^Il9ra^fl/fl^ mice. **(B)** Tfh cells (GFP^-^B220^-^CD4^+^CD44^+^CXCR5^+^PD-1^+^) and Tfr cells (GFP^+^B220^-^CD4^+^CD44^+^CXCR5^+^PD-1^+^) in the spleen, assessed using flow cytometry. Tfh cell percentages, *p = 0.0492 on day 7, *p = 0.0350 on day 35. Tfh cell numbers, *p = 0.0484 on day 7. **(C)** Expression levels of Bcl6 in Tfh cells. Histograms show the results obtained using intracellular flow cytometry. Graphs depict gMFI values as the levels of Bcl6 expression. ****p < 0.0001 on day 7, *p* = 0.0575 on day 35. **(D, E)** Analysis of IL-4^+^ Tfh cells and IL-21^+^ Tfh cells in the spleen cells. After stimulation with PMA and ionomycin for 6 hr, cells were examined using intracellular flow cytometry (**Supplementary Figure S5A**). **(D)** IL-4^+^ Tfh cells. Percentages, ****p < 0.0001; cell numbers, **p = 0.0050. **(E)** IL-21^+^ Tfh cells. Percentages, ***p = 0.0005; cell numbers, *p = 0.0162. **(F)** Analysis of Tfh cell populations. Tfh cells were grouped into a series of four populations (1-4 in the panels) based on the levels of PD-1 and CXCR5 expression in flow cytometric profiles. Graphs of %Tfh cells in each population are presented in the panels. From left to right, *p* = 0.0523 and **p = 0.0084 on day 7; *p* = 0.0962, *p = 0.0158, *p = 0.0311, and *p* = 0.0812 on day 35. Data represent the mean ± SD of 5-13 mice per group. Statistical significance was analyzed by the unpaired *t*-test. Similar results were obtained in three independent experiments.

Since Tfh cells cooperate with Tfr cells in the formation of GCs, we next investigated the impact of IL-9 on Tfh and Tfr cells (29). To separately examine Tfh and Tfr cells, experiments were conducted using FoxP3^GFP/DTR^CD4^Cre/+^Il9ra^fl/fl^ mice, following the same experimental protocol for CD4^Cre/+^Il9ra^fl/fl^ mice, as depicted in **Figure 2A** (**Figure 3A**). The level of Tfh cells (CD3^+^CD4^+^GFP^-^CXCR5^+^PD-1^+^) was markedly decreased in the spleen of FoxP3^GFP/DTR^CD4^Cre/+^Il9ra^fl/fl^ mice compared to control FoxP3^GFP/DTR^CD4^+/+^Il9ra^fl/fl^ mice (**Figure 3B**, **Supplementary Figure S4A**). In contrast, the level of Tfr cells (CD3^+^CD4^+^GFP^+^CXCR5^+^PD-1^+^) in the spleen of FoxP3^GFP/DTR^CD4^Cre/+^Il9ra^fl/fl^ mice was comparable to that in the control mice. Other CD4^+^ T cell subsets in the spleen of FoxP3^GFP/DTR^CD4^Cre/+^Il9ra^fl/fl^ mice were also comparable to those in the control mice (**Supplementary Figures S4B–G**). These results suggest that IL-9 had a regulatory effect mainly on Tfh cells in the formation of GCs, whereas its functional influence on Tfr cells and other CD4^+^ T cell subsets seemed to be limited. Following these observations, we studied the expression profiles of signature molecules characterizing Tfh cells. Remarkably, the level of Bcl6 in Tfh cells of FoxP3^GFP/DTR^CD4^Cre/+^Il9ra^fl/fl^ mice was significantly lower than that of the controls (**Figure 3C**). As the expression level of Bcl6 corresponds to the maturation state of Tfh cells, these findings suggest that IL-9 might regulate the development of Tfh cells within secondary lymphoid tissues in response to foreign antigens (30). The results also indicated a significant reduction in IL-4 or IL-21-expressing Tfh cells in FoxP3^GFP/DTR^CD4^Cre/+^Il9ra^fl/fl^ mice compared to the controls (**Figures 3D, E**, **Supplementary Figure S5A**). While the expression levels of PD-1 and ICOS were similar in Tfh cells of these two mouse groups, the level of CXCR5 was lower in Tfh cells of FoxP3^GFP/DTR^CD4^Cre/+^Il9ra^fl/fl^ mice (**Supplementary Figures S5B, C**). Collectively, these results imply the essential requirement of IL-9 for the emergence of fully functional Tfh cells. Considering the dynamic nature of Tfh cell status, CXCR5^hi^PD-1^hi^ Tfh cell populations are preferentially localized in GCs to foster GC B cells, while CXCR5^lo^PD-1^lo^ Tfh cell populations likely include developing and memory Tfh cells in interfollicular areas (31, 32). In this regard, CXCR5^+^PD-1^+^ Tfh cells, encompassing CXCR5^hi^PD-1^hi^ and CXCR5^lo^PD-1^lo^ Tfh cell populations, were subdivided into four sequential groups, and each group of Tfh cells was independently examined (**Figure 3F**). Tfh cells with higher expression levels of both CXCR5 and PD-1 tended to decrease in the initial phase, while this trend appeared to shift somewhat to CXCR5^lo^PD-1^lo^ Tfh cell populations in the recall phase (**Figure 3F**). These findings suggest the instructive role of IL-9 in Tfh cell regulation during the initial and recall phases, though its functional significance might not be identical across these phases.

### ILC2s as a chief IL-9 producer in the spleen

**Figure 4:**
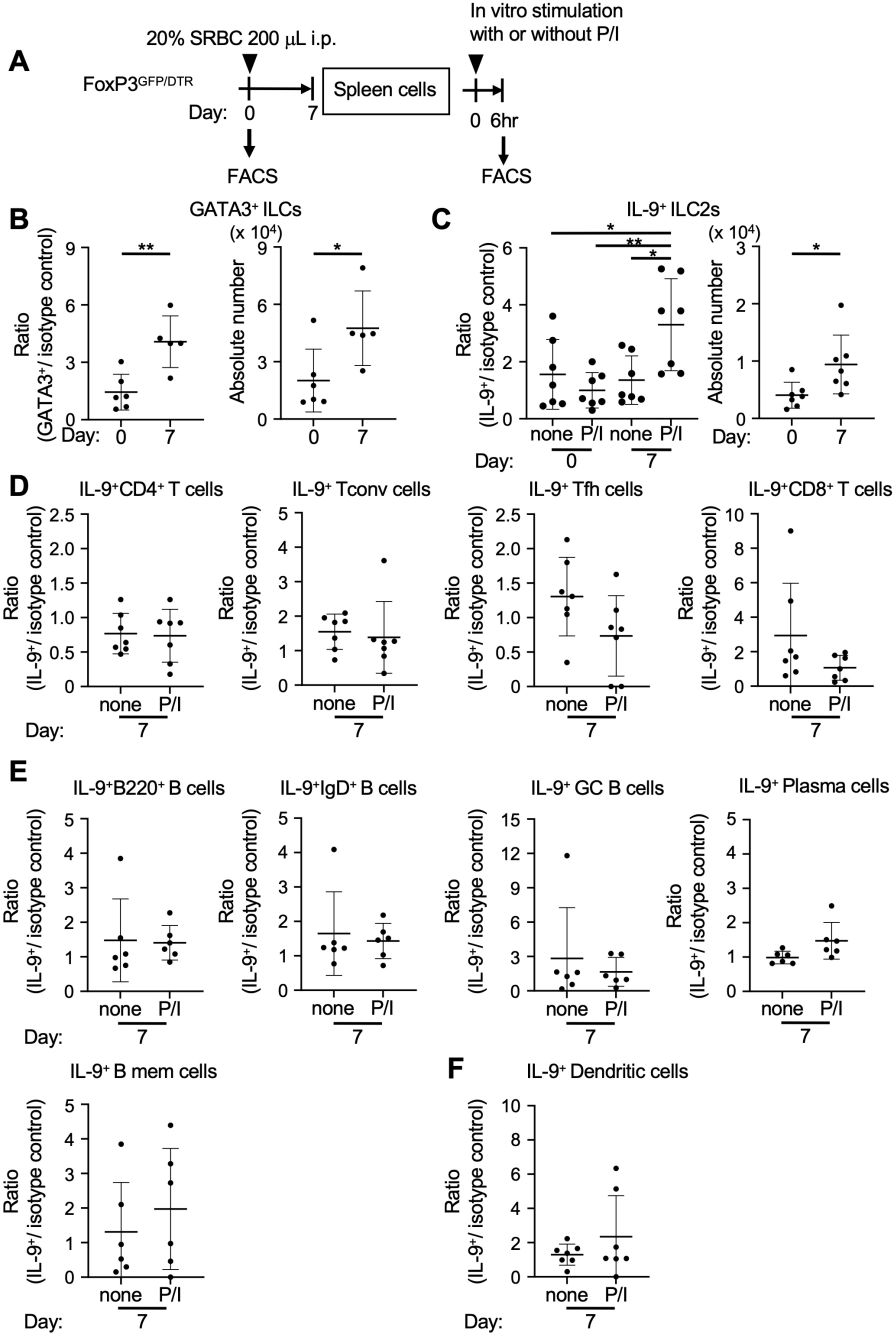
ILC2s produce IL-9 in secondary lymphoid tissues. **(A)** Experimental protocol for the immunization and analysis of FoxP3^GFP/DTR^ mice. Spleen cells were cultured with or without PMA and ionomycin (P/I) for 6 hr. **(B, C)** Analysis of ILCs (Lin^-^CD45^+^CD127^+^) and ILC2s (GATA3^+^ ILCs) in the spleen cells before and after immunization (day 0 and day 7, respectively), as measured using intracellular flow cytometry (**Supplementary Figures S6A–C**). **(B)** Ratios of ILC2s in ILCs (GATA3^+^/isotype control), **p = 0.0040. Cell numbers of ILC2s, *p = 0.0320. **(C)** Ratios of IL-9^+^ ILC2s in ILC2s (IL-9^+^/isotype control), *p = 0.0404 (none in day 0 vs. P/I in day 7), **p = 0.0048 (P/I of day 0 vs. P/I in day 7), *p = 0.0193 (none in day 7 vs. P/I in day 7). Cell numbers of IL-9^+^ ILC2s, *p = 0.0267. P/I, PMA and ionomycin. **(D–F)** Ratios (IL-9^+^/isotype control) of immune cells expressing IL-9, as measured using intracellular flow cytometry (**Supplementary Figures S7A–C**). **(D)** CD4^+^ T cells (CD3^+^CD4^+^) and CD4^+^ T subsets including Tconv cells (CD3^+^GFP^-^CD4^+^CXCR5^-^PD-1^-^), Tfh cells (CD3^+^GFP^-^CD4^+^CXCR5^+^PD-1^+^), and CD8^+^ T cells (CD3^+^CD8^+^). **(E)** B cells (B220^+^) and B cell subsets including IgD^+^ B cells (B220^+^IgD^+^), GC B cells (B220^+^IgD^-^FAS^+^GL7^+^), plasma cells (B220^-^IgD^-^CD138^+^), memory B cells (B220^+^IgD^-^FAS^+^GL7^-^CD38^+^). **(F)** Dendritic cells (CD11c^+^MHC-II^+^). Data represent the mean ± SD of 5-7 mice per group. Statistical significance was analyzed using Tukey’s multiple comparisons test (ratios in **C**) and the unpaired *t*-test (**B**, cell numbers in **C**, **D–F**). Similar results were obtained in three independent experiments.

We next focused on investigating a potential primary source of IL-9 within lymphoid tissues during immunizing conditions. To this end, FoxP3^GFP/DTR^ mice were intraperitoneally injected with SRBCs to analyze a range of immune cells in the spleen (**Figure 4A**). ILCs are considered a complementary part of the innate immune system, sharing evolutionarily conserved transcriptional programs with T cells, and reside in local tissues to maintain homeostasis and defend against pathogens. Group 2 ILCs (ILC2s) are capable of producing IL-9 in response to various stimuli, functioning as both an effector cytokine to surrounding cells and a survival factor for themselves (33). Our analysis revealed that ILC2s (GATA3^+^ ILCs) were significantly elevated in response to immunization, and IL-9-producing ILC2s (IL-9^+^GATA3^+^ ILCs) were also increased in parallel (**Figures 4B, C**, **Supplementary Figures S6A–C**). However, in our experimental procedures, we failed to observe the potential capacity to produce IL-9 in CD4^+^ T cells, including Tconv cells (CD3^+^GFP^-^CD4^+^CXCR5^-^PD-1^-^) and Tfh cells (CD3^+^GFP^-^CD4^+^CXCR5^+^PD-1^+^), as well as CD8^+^ T cells (CD3^+^CD8^+^; **Figure 4D**, **Supplementary Figure S7A**). Additionally, IL-9-producing cell populations were not clearly detected among B cell populations, including naive IgD^+^ B cells (B220^+^IgD^+^), GC B cells (B220^+^IgD^-^FAS^+^GL7^+^), memory B cells (B220^+^IgD^-^FAS^+^GL7^-^CD38^+^), and plasma cells (B220^-^IgD^-^CD138^+^; **Figure 4E**, **Supplementary Figure S7B**). Further analyses of dendritic cells (CD11c^+^MHC-II^+^) did not identify their capacity for IL-9 production (**Figure 4F**, **Supplementary Figure S7C**). Collectively, these findings suggest that ILC2s may be the primary producers of IL-9 in the spleen during antigen-specific humoral responses. Previous evidence indicates that CD4^+^ T cells could be modulated by ILC2s, possibly through their expression of major histocompatibility complex (MHC) class II and OX40 ligand (34, 35). In this context, the potential role of ILC2s might involve Tfh regulation to establish acquired humoral immunity. Given that the functional loss of IL-9 receptor in Tfh cells leads to a deficiency in antigen-specific antibody responses, a supportive microenvironment promoting Tfh cell activation might be maintained by IL-9-producing ILC2s in secondary lymphoid tissues.

### Follicular mantle zone B cells can help ILC2 activation

**Figure 5:**
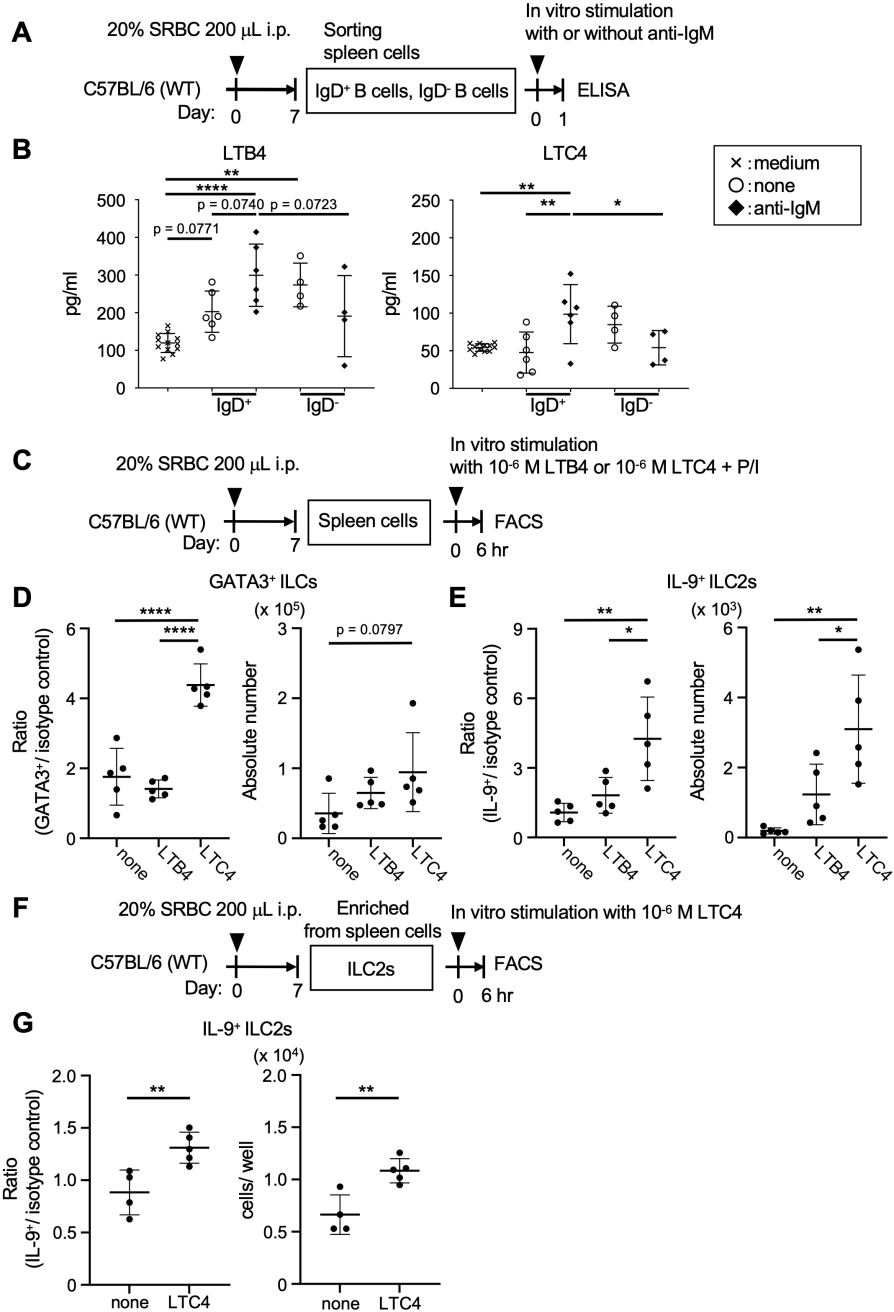
Follicular mantle zone B cells can activate ILC2s. **(A)** Experimental protocol for B cell analysis of wild-type (WT) C57BL/6 mice. After the immunization, IgD^+^ and IgD^-^ B cells were sorted from the spleen cells. These B cells were cultured with or without anti-IgM (20 μg/ml) for 24 hr. **(B)** Leukotrienes in the culture supernatants of B cells. The graphs for LTB4; *p* = 0.0740 (none vs. anti-IgM in IgD^+^ B cells), *p* = 0.0723 (IgD^+^ vs. IgD^-^ B cells with anti-IgM), *p* = 0.0771 (medium vs. none in IgD^+^ B cells), **p = 0.0015 (medium vs. none in IgD^-^ B cells), ****p < 0.0001 (medium vs. anti-IgM in IgD^+^ B cells). The graphs for LTC4; **p = 0.0071 (none vs. anti-IgM in IgD^+^ B cells), *p = 0.0493 (IgD^+^ vs. IgD^-^ B cells with anti-IgM), **p = 0.0068 (medium vs. anti-IgM in IgD^+^ B cells). **(C)** Experimental protocol for ILC analysis of the WT mice. After the immunization, spleen cells were cultured with or without leukotriene (LTB4 or LTC4) and PMA and ionomycin (P/I) for 6 hr. **(D)** Ratios of ILC2s in ILCs (GATA3^+^/isotype control), ****p < 0.0001 (none vs. LTC4), ****p < 0.0001 (LTB4 vs. LTC4). Cell numbers of ILC2s, *p* = 0.0797 (none vs. LTC4). **(E)** Ratios of IL-9^+^ ILC2s in ILC2s (IL-9^+^/isotype control), **p = 0.0025 (none vs. LTC4), *p = 0.0152 (LTB4 vs. LTC4). Cell numbers of IL-9^+^ ILC2s, **p = 0.0020 (none vs. LTC4), *p = 0.0345 (LTB4 vs. LTC4). **(F)** Experimental protocol for ILC2 analysis of the WT mice. After the immunization, ILC2s were isolated from spleen cells and cultured with or without LTC4 for 6 hr. **(G)** Ratios of IL-9^+^ ILC2s in ILC2s (IL-9^+^/isotype control), *p = 0.0094. Cell numbers of IL-9^+^ ILC2s, **p = 0.0044. Data represent the mean ± SD of 3-7 mice per group. Statistical significance was analyzed using Tukey’s multiple comparisons test **(B, D, E)** and the unpaired *t*-test **(G)**. Similar results were obtained in two independent experiments.

Following these results, we further explored the mechanism by which ILC2s potentiate Tfh cell activation in lymphoid tissues to achieve specific humoral immune responses. ILC2s express multiple cell-surface molecules, including various cytokine receptors, killer cell lectin-like receptor G1 (KLRG1), receptors for lipid mediators such as S1P and leukotrienes, neuropeptide receptors, and a thymus stromal lymphopoietin (TSLP) receptor (24, 36). Generally, lipid mediators ensure the rapid activation of inflammatory and immune responses. Importantly, ILC2s can migrate via S1P-mediated chemotaxis, a mechanism shared with Tfh cells, and these navigation cues enable access to the T-B border and GC B cells (5, 37, 38). This implies a potential role of B cells in directing the tropism of ILC2s and Tfh cells. As a part of the T-B border, follicular mantle zones surrounding GCs preferentially harbor IgD^+^ B cells, which are noted to highly express arachidonate 5-lipoxygenase (Alox5) as a rate-limiting enzyme in leukotriene production (25–27). Upon cell activation through the engagement of B cell receptors (BCRs), IgD^+^ B cells could produce Alox5-related leukotrienes including LTB4 and LTC4, as suggested by *in vitro* experiments using sorted splenocytes derived from SRBC-immunized wild-type mice (**Figures 5A, B**). Without the stimulation of BCRs, IgD^-^ B cells produced LTB4, but not LTC4. Specifically, LTC4, a cysteinyl leukotriene, could induce the production of IL-9 in ILC2s, as demonstrated by experiments using the total splenocytes from the immunized mice (**Figures 5C–E**). Following these results, ILC2s derived from spleen cells using a mouse ILC2 enrichment kit contained cells with the capacity to produce IL-9 in response to LTC4, as assessed by intracellular FACS analysis (**Figures 5F, G**, **Supplementary Figures S8A, B**). Further experiments using MD4 transgenic mice, which possess B cells expressing IgD and IgM specific to HEL, were conducted to examine this mechanism. HEL exposure *in vivo* resulted in a marked elevation of IL-9-producing ILC2s (GATA3^+^ ILCs), while ILC3s (RORγt^+^ ILCs) remained unaffected (**Supplementary Figures S8C, D**). These findings suggest a potential B-cell-associated mechanism for IL-9 production by ILC2s, which might contribute to maintaining the functional integrity of Tfh cells in organized lymphoid tissues.

### Pathological role of IL-9-activated Tfh cells

**Figure 6:**
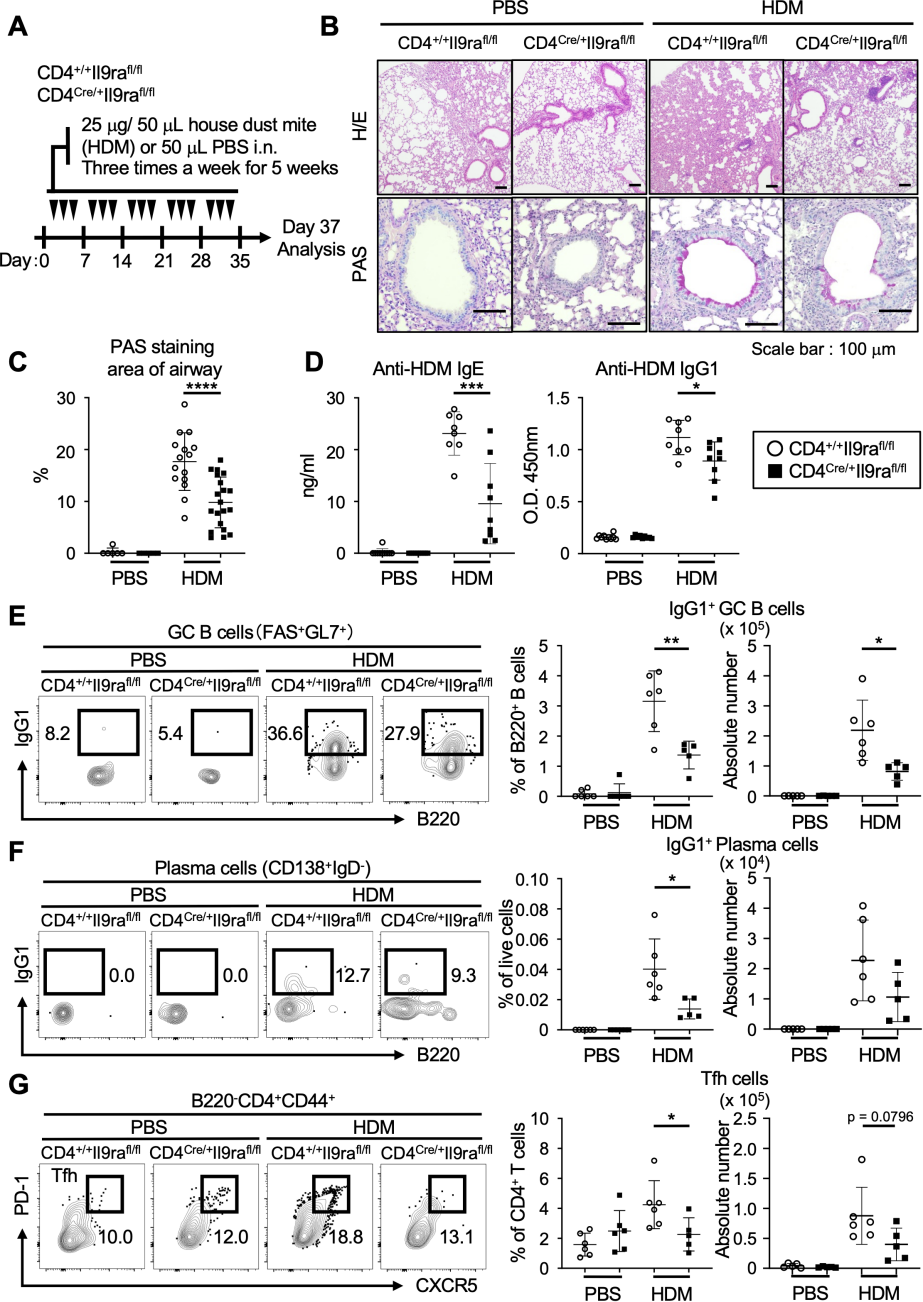
IL-9 promotes Tfh cells to generate allergen-specific antibodies. **(A)** Experimental protocol for HDM exposure in CD4^Cre/+^Il9ra^fl/fl^ and control CD4^+/+^Il9ra^fl/fl^ mice. Mice were sensitized by intranasal instillation of 25 μg HDM in 50 μl PBS or 50 μl PBS alone as a control for three days per week for 5 consecutive weeks. The mice were analyzed on day 37. **(B)** Representative microscopy images of formalin-fixed paraffin-embedded tissue sections of the lung stained with HE and PAS. **(C)** Quantification of PAS-positive mucous cells in airways. Graphs depict the ratio of PAS^+^ areas within the airway area for each group. Imaging data were analyzed using ImageJ software. ****p < 0.0001. **(D)** Serum levels of HDM-specific antibodies. Anti-HDM-specific IgE, ***p = 0.0005; anti-HDM-specific IgG1, *p = 0.0181. **(E–G)** Representative flow cytometric profiles and graphs of lymphocyte subsets within the mediastinal lymph nodes (**Supplementary Figures S3A, B**). **(E)** IgG1^+^ GC B cells (B220^+^IgD^-^FAS^+^GL7^+^). Percentages, **p = 0.0055; cell numbers, *p = 0.0167. **(F)** IgG1^+^ plasma cells (B220^lo^IgD^-^CD138^hi^). Percentages, *p = 0.0205. **(G)** Tfh cells (B220^-^CD4^+^CD44^+^CXCR5^+^PD-1^+^). Percentages, *p = 0.0446; cell numbers, *p* = 0.0796. Data represent the mean ± SD of 5-10 mice per group. Statistical significance was analyzed using the unpaired *t*-test **(C–G)**. Similar results were obtained in three independent experiments.

While IL-9 is considered a pleiotropic cytokine, it is implicated in the production of IgE and IgG during allergic inflammation (16, 18, 39). Expanding this notion, our subsequent investigation focused on the pathological relevance of IL-9 in relation to the production of allergen-specific antibodies. We analyzed an allergic asthma model induced by HDM, a common clinical allergen known to cause bronchial asthma through type 2 inflammation (40). Repetitive intranasal challenges of HDM for 5 weeks resulted in chronic airway inflammation with goblet cell hyperplasia in control CD4^+/+^Il9ra^fl/fl^ mice, while CD4^Cre/+^Il9ra^fl/fl^ mice showed relative resistance to airway inflammation (**Figures 6A–C**). Of note, the serum levels of anti-HDM IgE and anti-HDM IgG1 antibodies were markedly reduced in CD4^Cre/+^Il9ra^fl/fl^ mice compared to controls, suggesting a diminished ability to produce HDM-specific antibodies in CD4^Cre/+^Il9ra^fl/fl^ mice (**Figure 6D**). Consistent with these observations, analysis of cell populations in the mediastinal (draining) lymph nodes revealed lower levels of IgG1-presenting GC B cells (IgG1^+^FAS^+^GL7^+^) and plasma cells (IgG1^+^IgD^-^CD138^+^) in CD4^Cre/+^Il9ra^fl/fl^ mice compared to controls (**Figures 6E, F**, **Supplementary Figures S9A–D**). These findings were likely linked to the reduced proportion of Tfh cells (B220^-^CD4^+^CD44^+^CXCR5^+^PD-1^+^) residing in the mediastinal lymph nodes of CD4^Cre/+^Il9ra^fl/fl^ mice (**Figure 6G**, **Supplementary Figure S9E**). Overall, these results highlight the crucial role of IL-9 receptor signaling in enabling Tfh cells to promote allergen-specific humoral responses.

## Discussion

In this study, we show that IL-9 receptor signaling in Tfh cells plays an important role in promoting the development of GC B cells and the production of high-affinity antibodies. IL-9 has diverse roles, ranging from protective immunity to immunopathology, depending on the specific setting and microenvironment. However, the role of IL-9 in regulating Tfh cells has not yet been fully elucidated. Our findings indicate that human and mouse Tfh cells express IL-9 receptor, and IL-9 receptor signaling potentiates Bcl6 expression in Tfh cells, ensuring their functional integrity. Considering the limited capacity of IL-9 receptor signaling in Tfr cells, IL-9 may facilitate the formation of GCs based on the functional balance of Tfh cells and Tfr cells. Studies using immunization models with foreign antigens, such as SRBC and HDM, suggest that the IL-9-dependent mechanism regulating Tfh cells may be common in acquired humoral responses overall. This mechanism does not seem to be specific to type 2 inflammation, which is marked by type 2 cytokines like IL-4, IL-5, IL-9, and IL-13, hallmarks of allergic reactions (41). Further studies would clarify the potential functional bias in Tfh cell regulation by IL-9 in a type 2 inflammatory environment. IL-9 receptor signaling modulates immune responses and inflammatory reactions by controlling various cellular processes through the activation of JAK-STAT pathways, particularly associated with STAT1, STAT3, and STAT5 (13). During the differentiation of Tfh cells, STAT1, STAT3, and STAT4 induce the expression of Bcl6, which in turn promotes IL-4 and IL-21 production and CXCR5 expression (42). In addition to the JAK-STAT pathways, IL-9 receptor signaling also activates the PI3K pathway, leading to enhanced Bcl6 expression in Tfh cells (43). Thus, multiple signals from IL-9 receptor may contribute to Bcl6 induction in Tfh cells for the efficient formation of GCs. In the future, a comprehensive analysis of IL-9 receptor signaling could uncover additional regulatory mechanisms governing Tfh cells.

In secondary lymphoid tissues, Tfh cells undergo multiple differentiation steps across various anatomical sites. Initially, naive CD4^+^ T cells in T zones are primed by antigen-presenting dendritic cells for early Tfh differentiation, supported by co-stimulatory molecules and cytokines provided by dendritic cells in an MHC class II-dependent manner (1, 2, 42). Along with the expression of ICOS, Tfh cells tend to express specific receptors such as CXCR5 and S1PR2 (5). Subsequently, Tfh cells are guided by CXCL13 (a ligand of CXCR5) and S1P to migrate preferentially around the T-B border, where they can be activated by B cells bearing the appropriate antigen under the cognate interaction. The T-B interaction is essential for the maturation of Tfh cells through the stimulation by cytokines such as IL-4 and IL-21 and cell surface molecules including CD80, CD86, and CD40L, as well as T-cell receptor signals, advancing Tfh cell function to form GCs. Experimental findings suggest that IL-9 is dispensable for the expression of ICOS and PD-1 in Tfh cells but augments Tfh cell function, indicating a potential cardinal role for IL-9 during Tfh differentiation. As IL-9 receptor signaling is likely related to the upregulation of CXCR5, and Tfh cells were detected in GCs to a lesser extent in CD4^Cre/+^Il9ra^fl/fl^ mice, it is possible that IL-9 supports Tfh cell function prior to the process of GC formation. In this context, sites within lymphoid tissues that facilitate Tfh development might be considered as possible effector areas for supplying IL-9 to Tfh cells. Further studies are needed to carefully consider the step(s) requiring the strength of IL-9 receptor signaling to foster Tfh cell differentiation in and outside secondary lymphoid tissues (3, 44). These studies will include the analysis of the expression profiles of functional molecules such as CD40L, TIGIT, and LAG3 in relation to IL-9 receptor signaling.

ILCs are found in various tissues and serve as functional sentinels that detect invading pathogens and tissue damage. They have the ability to promptly release specific factors to modulate immune responses. Activated ILC2s produce Th2 cytokines such as IL-4, IL-5, IL-9, and IL-13, among which IL-9 immensely contributes to the survival of ILC2s, and IL-13 is required for the induction of CD11c^+^MHC class II^hi^ dendritic cells (24, 25, 33). Recently, ILC2s have been identified in interfollicular areas of lymphoid tissues, suggesting their interaction with T cells (45). Since the number of IL-9-producing ILCs significantly exceeds that of IL-9-producing T cells, ILC2s may play an integral part in coordinating innate and adaptive immunity through an IL-9-mediated mechanism (33, 46). In addition, ILC2s can promote the differentiation of naive CD4^+^ T cells, and the absence of IL-9 receptors results in T-cell dysfunction, further implying the cardinal role of IL-9 in the regulation of T cells (47, 48). A follicular regulatory ILC population has recently been reported in GCs of tonsils and lymph nodes, but it is still unclear whether they produce IL-9 (49). The impact of inflammatory or induced ILC2s in non-lymphoid tissues such as the lung, intestine, and skin on Tfh cells in the draining or distantly related lymph nodes is an intriguing research area. Further studies using IL-9 reporter mice, for example, will allow for the analysis of the functional association of ILC2s and Tfh cells, as well as the verification of potential IL-9 production by other cells in the immune or inflammatory environments.

This study focused on investigating the potential relationship between ILC2s and follicular mantle zone B cells to address the IL-9-dependent regulation of Tfh cells. Antigen-bound cognate B cells positioned around the T-B border, comprised by mantle zones, can produce Alox5-related lipid mediators such as LTB4 and cysteinyl leukotrienes, potentially influencing the capacity of ILC2s to produce IL-9. Experimental evidence suggests that Alox5-deficient mice exhibit fewer GCs with developmental insufficiency of Tfh cells (27). Noncognate B cells in mantle zones can also generate these lipid mediators upon antigen binding, potentially affecting Tfh cell maturation by stimulating IL-9-producing ILC2s. This conditioning may increase the activation of Tfh cells, serving as a link connecting innate immune responses to specific humoral responses. Of note, a subset of the Tfh cells is capable of producing IL-9, suggesting a potential autocrine mechanism within this subset (12). Memory B cells can also produce IL-9, promoting Tfh cell maturation for GC formation (19). Future research focusing on the activation of Tfh cells in collaboration with IL-9-producing ILC2s and follicular mantle zone B cells is anticipated to enhance our understanding of Tfh cell biology.

In summary, we report the pivotal role of IL-9 in Tfh cell development, likely supported by IL-9-producing ILC2s, which may engage in the interaction between antigen-stimulated B cells and Tfh cell maturation. Understanding the tissue microenvironments that influence Tfh cell programming is crucial, as Tfh cell dysregulation can contribute to allergies, autoimmune diseases, and other inflammatory conditions. ILC2s play a multifaceted role in the immune system, influencing various physiological and pathological processes. Integrating ILC2s into the current understanding of Tfh cell biology could be beneficial, including their lineage differentiation, effector function, memory potential, and plasticity.

## Data Availability

The original contributions presented in the study are included in the article/**Supplementary Material**. Further inquiries can be directed to the corresponding author.

## Supplementary material

Supplementary Figure 1 Establishment of CD4^Cre/+^Il9ra^fl/fl^ and CD4^+/+^Il9ra^fl/fl^ mice. **(A)** Experimental protocol for the immunization of CD4^Cre/+^Il9ra^fl/fl^ and control CD4^+/+^Il9ra^fl/fl^ mice. **(B)** Gating strategy and sorting of Tfh cells (CD44^+^CXCR5^+^PD-1^+^) and Tnaiv cells (CD44^-^CXCR5^-^PD-1^-^) from splenocytes. Graphs show the expression levels of *Il9ra* transcripts in Tfh cells and Tnaiv cells as assessed using RT-qPCR. **(C)** Gating strategy and sorting of naive B cells (Bnaiv; B220^+^IgD^+^) and memory B cells (Bmem; B220^+^IgD^-^FAS^+^GL7^-^CD38^+^). Graphs show the expression levels of *Il9ra* transcripts in Bnaiv and Bmem cells as assessed using RT-qPCR. Data represent the mean ± SD of 3-6 mice per group. *****p* < 0.0001 (CD4^+/+^Il9ra^fl/fl^ Tnaiv vs. CD4^+/+^Il9ra^fl/fl^ Tfh, CD4^+/+^Il9ra^fl/fl^ Tfh vs. CD4^Cre/+^Il9ra^fl/fl^ Tfh). ***p* = 0.0011 (CD4^+/+^Il9ra^fl/fl^ Bnaiv vs. CD4^+/+^Il9ra^fl/fl^ Bmem). Statistical analysis was performed using Tukey’s multiple comparisons test. Similar results were obtained in three independent experiments.

Supplementary Figure 2 Analyses of thymocytes and splenocytes of CD4^Cre/+^Il9ra^fl/fl^ and CD4^+/+^Il9ra^fl/fl^ mice in steady state. **(A)** Gating strategy for thymocytes. **(B)** Percentages of thymocyte populations per live cells, including double negative (DN, CD4^-^CD8^-^) cells, double positive (DP, CD4^+^CD8^+^) T cells, single positive CD4^+^CD8^-^ T cells, and CD4^-^CD8^+^ T cells. **(C)** Gating strategy for spleen cells. **(D)** Percentages of immune cells per total cells, including CD11c^+^MHC-II^+^ dendritic cells, B220^+^ B cells, CD4^+^CD8^-^ T cells, and CD4^-^CD8^+^ T cells. Data represent the mean ± SD of 7-8 mice per group. Statistical significance was determined using the unpaired *t*-test. Similar results were obtained in two independent experiments.

Supplementary Figure 3 Analyses of B and T cells in CD4^Cre/+^Il9ra^fl/fl^ and CD4^+/+^Il9ra^fl/fl^ mice (corresponding to **Figures 2**, **6**). **(A)** Gating strategy for B cell populations. **(B)** Gating strategy for T cell populations. **(C)** Percentages and numbers of CD4^+^ T cells. Data represent the mean ± SD of 7-9 mice per group. Statistical significance was determined using the unpaired *t*-test. Similar results were obtained in three independent experiments.

Supplementary Figure 4 Analyses of CD4^+^ T cells in FoxP3^GFP/DTR^CD4^Cre/+^Il9ra^fl/fl^ and FoxP3^GFP/DTR^CD4^+/+^Il9ra^fl/fl^ mice (corresponding to **Figure 3**). **(A)** Gating strategy for CD4^+^ T cell populations. (B-G) Percentages and numbers of each CD4^+^ T cell population. **(B)** CD4^+^T cells. **(C)** Tnaiv cells. **(D)** Tconv cells. **(E)** PD-1^+^ Tconv cells. **(F)** Treg cells. **(G)** PD-1^+^ Treg cells. Data represent the mean ± SD of 10-13 mice per group. Statistical significance was analyzed by the unpaired *t*-test. Similar results were obtained in three independent experiments.

Supplementary Figure 5 Analyses of Tfh cells of FoxP3^GFP/DTR^CD4^Cre/+^Il9ra^fl/fl^ and FoxP3^GFP/DTR^ CD4^+/+^Il9ra^fl/fl^ mice (corresponding to **Figure 3**). **(A)** Representative FACS plots of IL-4^+^ Tfh cells and IL-21^+^ Tfh cells. **(B)** Representative histograms of CXCR5, PD-1, and ICOS expression in Tfh cells. **(C)** Expression levels of CXCR5, PD-1, and ICOS in Tfh cells according to gMFI values. **p* = 0.0269. Data show the mean ± SD of 7-10 mice per group. Statistical significance was analyzed by the unpaired *t*-test. Similar results were obtained in three independent experiments.

Supplementary Figure 6 Gating strategy for ILCs in FoxP3^GFP/DTR^ mice (corresponding to **Figure 4**). **(A)** Gating strategy for ILCs. Lineage (Lin) markers include B220, CD3, CD11b, CD11c, Gr-1, NK1.1, TCRβ, TCRγδ, and TER119. **(B)** Representative FACS plots of GATA3^+^ ILCs (ILC2s) compared with isotype control. **(C)** Representative FACS plots of IL-9^+^ ILC2s compared with isotype control.

Supplementary Figure 7 Gating strategy for immune cell populations in FoxP3^GFP/DTR^ mice (corresponding to **Figure 4**). **(A)** Gating strategy for T cell populations. **(B)** Gating strategy for B cell populations. **(C)** Gating strategy for dendritic cells.

Supplementary Figure 8 Analyses of ILCs in wild-type and MD4 mice (corresponding to **Figure 5**). **(A)** Representative FACS plots of ILCs concerning the reanalysis of cells obtained from splenocytes using a mouse ILC2 enrichment kit. Lineage markers are represented as Lin; B220, CD3, CD11b, CD11c, Gr-1, NK1.1, TCRβ, TCRγδ, and TER119. **(B)** %ILCs in A. Each dot represents the average value of a group of mice (n = 3-4). *****p* < 0.0001. Data represent the mean ± SD of 4-5 groups. **(C)** Experimental protocol for immunization of MD4 mice. 100 μg HEL/CFA was administered intraperitoneally. After the immunization, spleen cells were cultured with or without P/I for 6 hr. **(D)** Percentages of GATA3^+^ ILCs (ILC2s) in splenocytes, **p* = 0.0490. Percentages of IL-9^+^ ILC2s in ILC2s, **p* = 0.0317. **(E)** Percentages of RORgt ILCs (ILC3s) in splenocytes and IL-9^+^ ILC3s in ILCs. Data represent the mean ± SD of 5 mice per group. Statistical significance was analyzed using the unpaired *t*-test. Similar results were obtained in two independent experiments.

Supplementary Figure 9 Analysis of immune cells in mediastinal lymph nodes in HDM-induced allergic asthma models (corresponding to **Figure 6**). **(A)** Numbers of live cells in mediastinal lymph nodes, *p* = 0.0507. **(B–E)** Percentages and numbers of cell populations in mediastinal lymph nodes (**Supplementary Figures 3A, B**). **(B)** B220^+^ B cells. Percentages, ***p* = 0.0049; cell numbers, *p* = 0.0614. **(C)** GC B cells. Cell numbers, **p* = 0.0264. **(D)** Plasma cells. **(E)** CD4^+^ T cells. Data represent the mean ± SD of 5-6 mice per group. Statistical significance was analyzed using the unpaired *t*-test **(A–E)**. All data are from CD4^+/+^Il9ra^fl/fl^ (open circle) and CD4^Cre/+^Il9ra^fl/fl^ mice (filled square). Similar results were obtained in three independent experiments.
