# Supplementary figures and images for: Interleukin 9 mediates T follicular helper cell activation to promote antibody responses
Source: Front Immunol. 2024 Sep 30;15:1441407. doi: 10.3389/fimmu.2024.1441407 (PMC11471543; doi:10.3389/fimmu.2024.1441407)

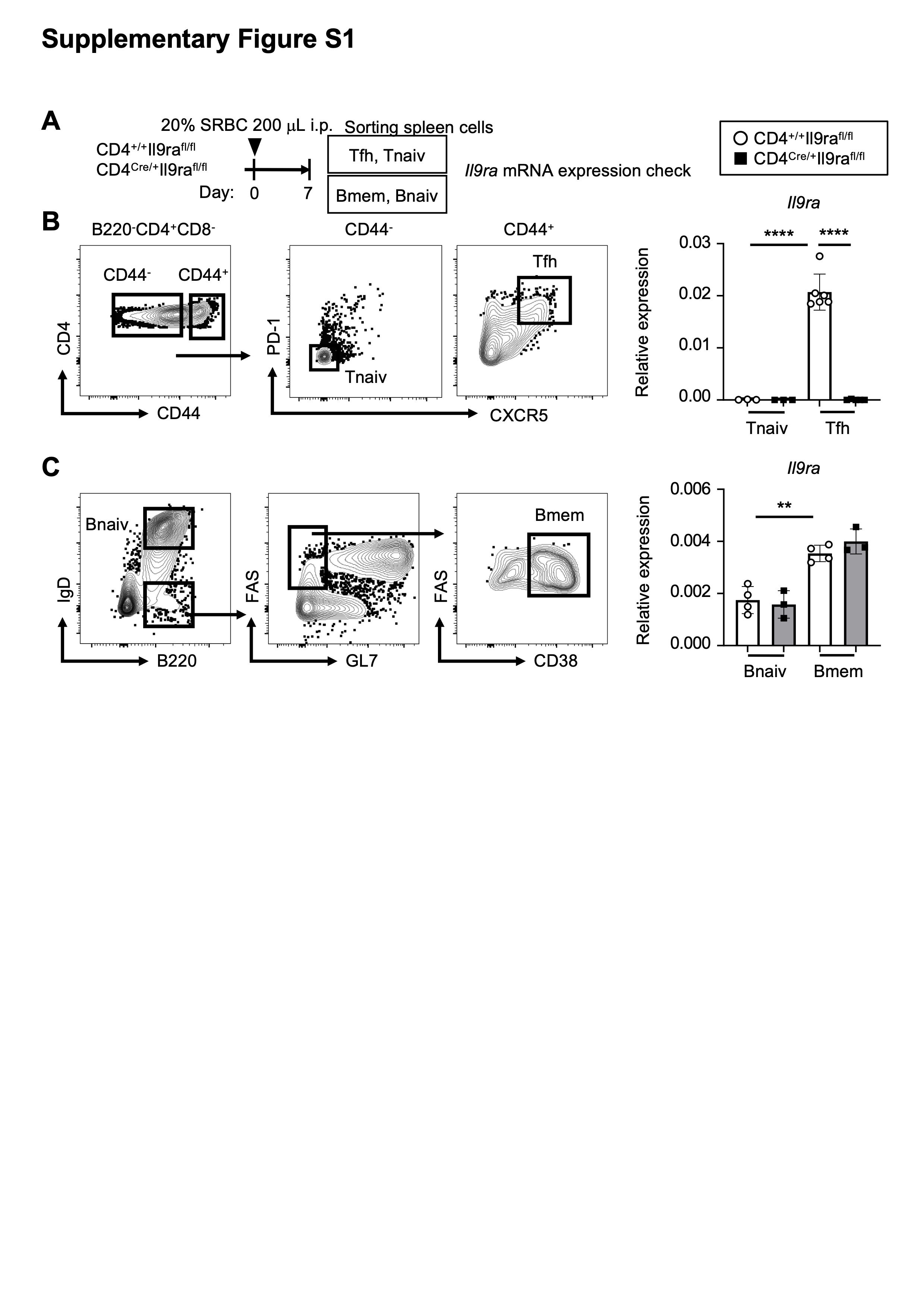

Supplement: Supplementary Figure 1 — Establishment of CD4Cre/+Il9rafl/fl and CD4+/+Il9rafl/fl mice. (A) Experimental protocol for the immunization of CD4Cre/+Il9rafl/fl and control CD4+/+Il9rafl/fl mice. (B) Gating strategy and sorting of Tfh cells (CD44+CXCR5+PD-1+) and Tnaiv cells (CD44-CXCR5-PD-1-) from splenocytes. Graphs show the expression levels of Il9ra transcripts in Tfh cells and Tnaiv cells as assessed using RT-qPCR. (C) Gating strategy and sorting of naive B cells (Bnaiv; B220+IgD+) and memory B cells (Bmem; B220+IgD-FAS+GL7-CD38+). Graphs show the expression levels of Il9ra transcripts in Bnaiv and Bmem cells as assessed using RT-qPCR. Data represent the mean ± SD of 3-6 mice per group. ****p < 0.0001 (CD4+/+Il9rafl/fl Tnaiv vs. CD4+/+Il9rafl/fl Tfh, CD4+/+Il9rafl/fl Tfh vs. CD4Cre/+Il9rafl/fl Tfh). **p = 0.0011 (CD4+/+Il9rafl/fl Bnaiv vs. CD4+/+Il9rafl/fl Bmem). Statistical analysis was performed using Tukey’s multiple comparisons test. Similar results were obtained in three independent experiments. [file Image1.jpeg]

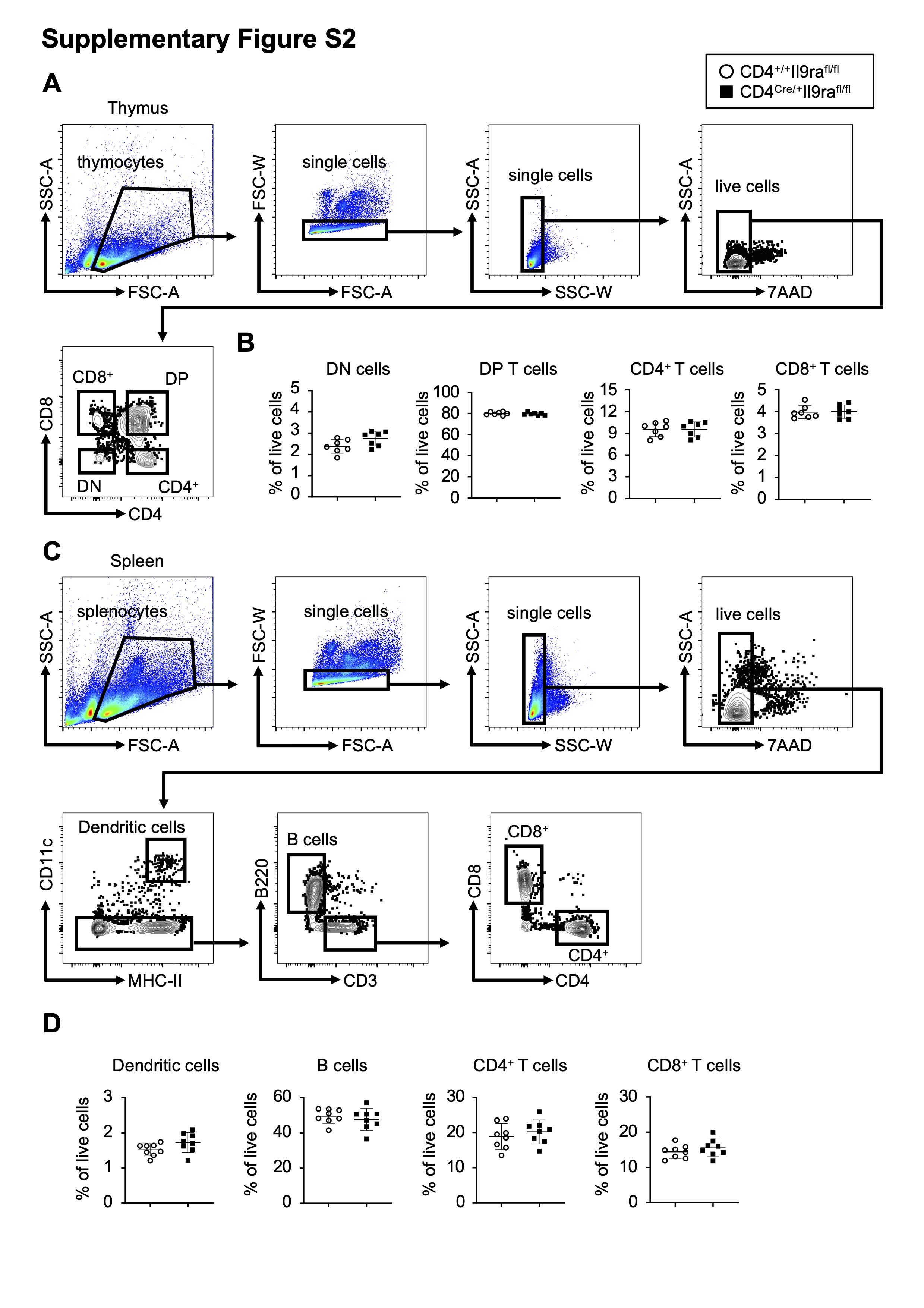

Supplement: Supplementary Figure 2 — Analyses of thymocytes and splenocytes of CD4Cre/+Il9rafl/fl and CD4+/+Il9rafl/fl mice in steady state. (A) Gating strategy for thymocytes. (B) Percentages of thymocyte populations per live cells, including double negative (DN, CD4-CD8-) cells, double positive (DP, CD4+CD8+) T cells, single positive CD4+CD8- T cells, and CD4-CD8+ T cells. (C) Gating strategy for spleen cells. (D) Percentages of immune cells per total cells, including CD11c+MHC-II+ dendritic cells, B220+ B cells, CD4+CD8- T cells, and CD4-CD8+ T cells. Data represent the mean ± SD of 7-8 mice per group. Statistical significance was determined using the unpaired t-test. Similar results were obtained in two independent experiments. [file Image2.jpeg]

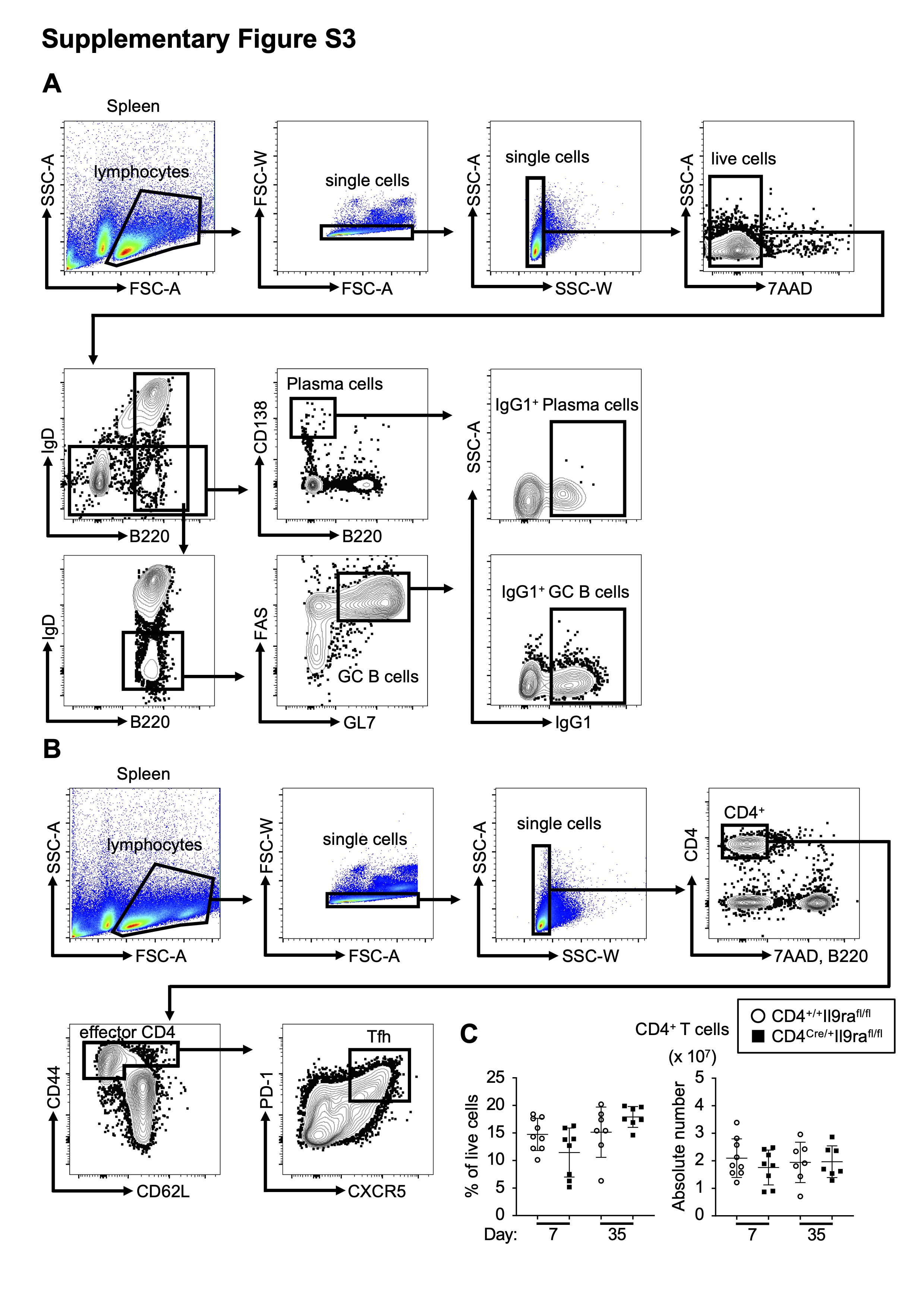

Supplement: Supplementary Figure 3 — Analyses of B and T cells in CD4Cre/+Il9rafl/fl and CD4+/+Il9rafl/fl mice (corresponding to Figures 2 , 6 ). (A) Gating strategy for B cell populations. (B) Gating strategy for T cell populations. (C) Percentages and numbers of CD4+ T cells. Data represent the mean ± SD of 7-9 mice per group. Statistical significance was determined using the unpaired t-test. Similar results were obtained in three independent experiments. [file Image3.jpeg]

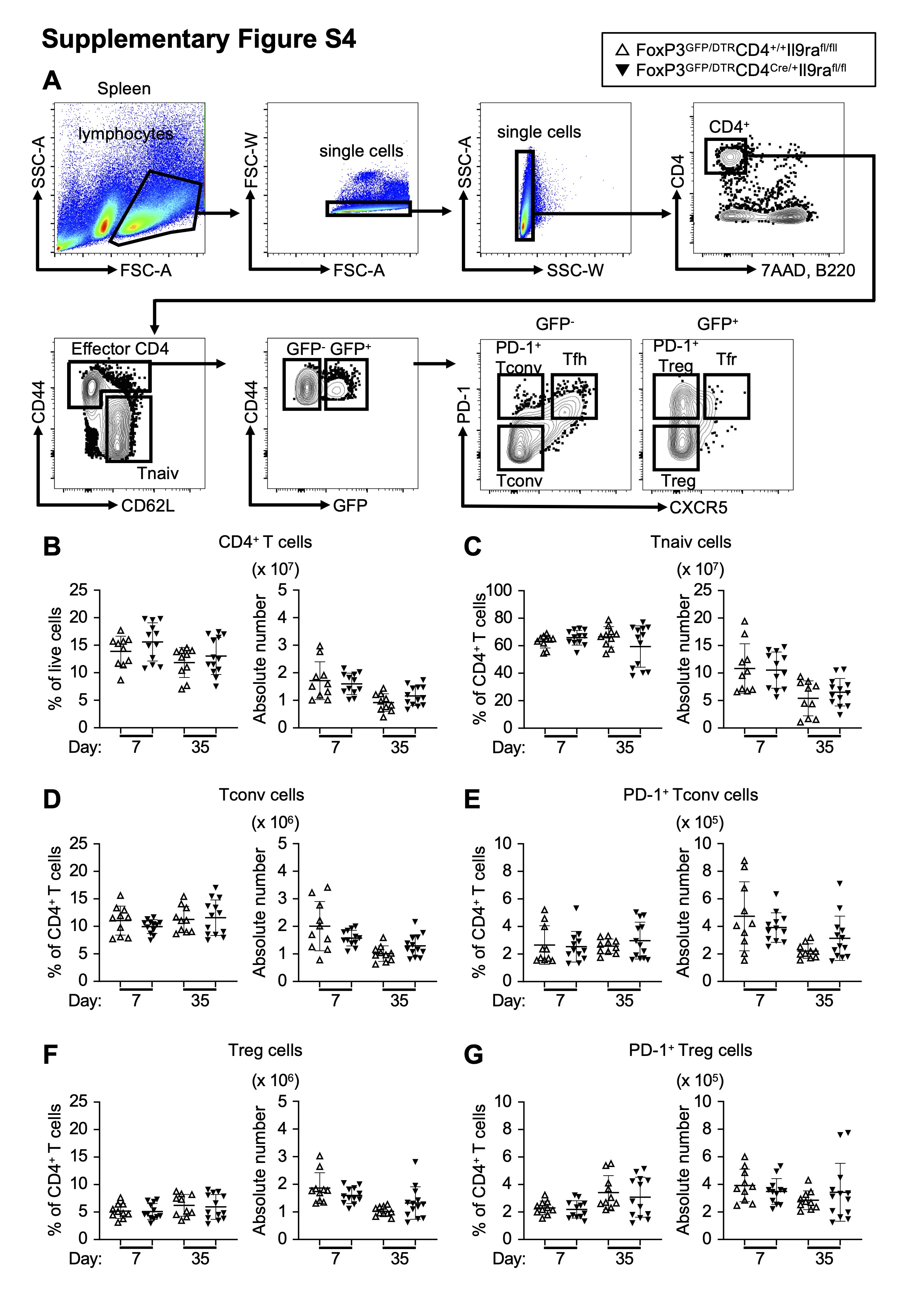

Supplement: Supplementary Figure 4 — Analyses of CD4+ T cells in FoxP3GFP/DTRCD4Cre/+Il9rafl/fl and FoxP3GFP/DTRCD4+/+Il9rafl/fl mice (corresponding to Figure 3 ). (A) Gating strategy for CD4+ T cell populations. (B-G) Percentages and numbers of each CD4+ T cell population. (B) CD4+T cells. (C) Tnaiv cells. (D) Tconv cells. (E) PD-1+ Tconv cells. (F) Treg cells. (G) PD-1+ Treg cells. Data represent the mean ± SD of 10-13 mice per group. Statistical significance was analyzed by the unpaired t-test. Similar results were obtained in three independent experiments. [file Image4.jpeg]

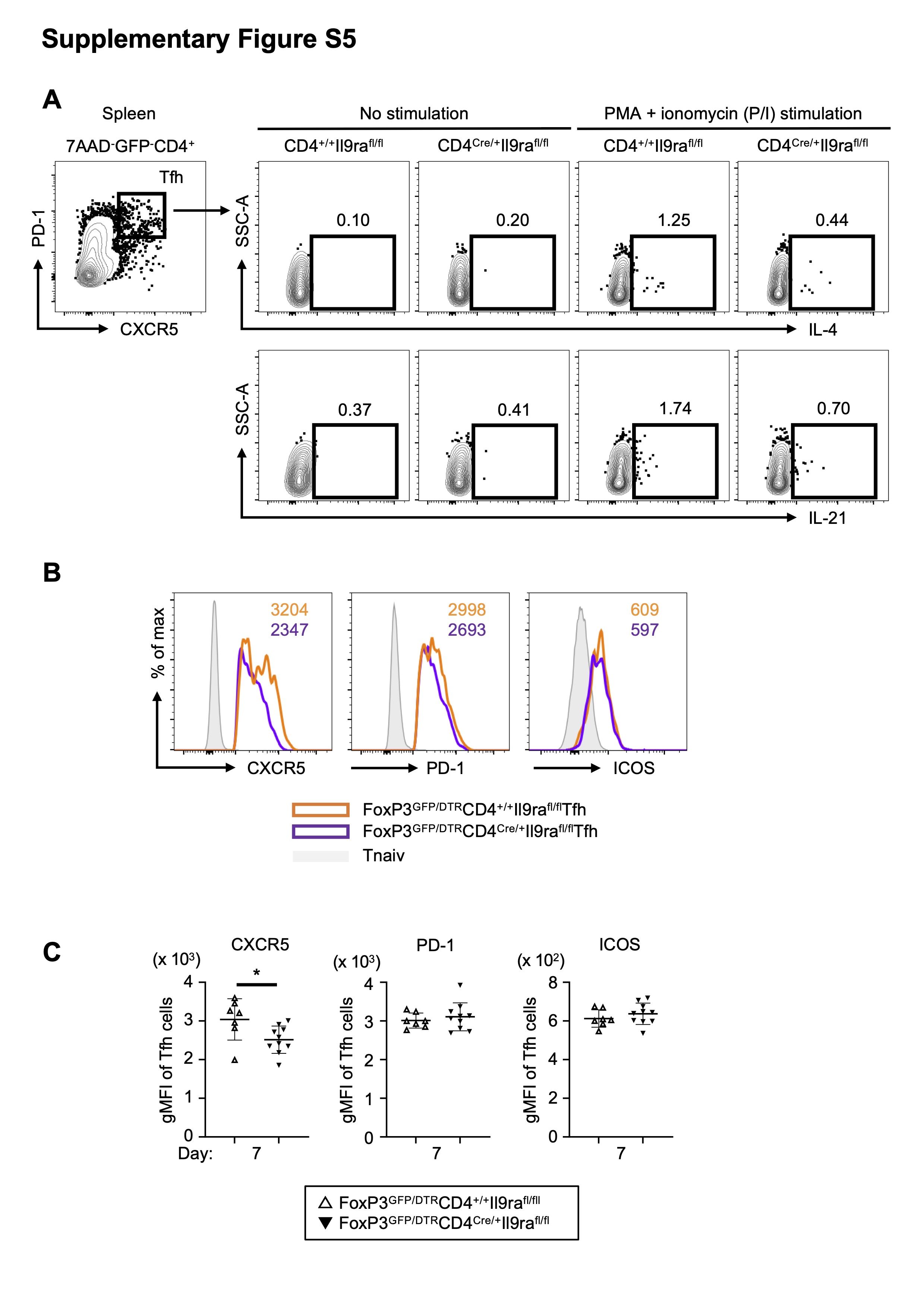

Supplement: Supplementary Figure 5 — Analyses of Tfh cells of FoxP3GFP/DTRCD4Cre/+Il9rafl/fl and FoxP3GFP/DTR CD4+/+Il9rafl/fl mice (corresponding to Figure 3 ). (A) Representative FACS plots of IL-4+ Tfh cells and IL-21+ Tfh cells. (B) Representative histograms of CXCR5, PD-1, and ICOS expression in Tfh cells. (C) Expression levels of CXCR5, PD-1, and ICOS in Tfh cells according to gMFI values. *p = 0.0269. Data show the mean ± SD of 7-10 mice per group. Statistical significance was analyzed by the unpaired t-test. Similar results were obtained in three independent experiments. [file Image5.jpeg]

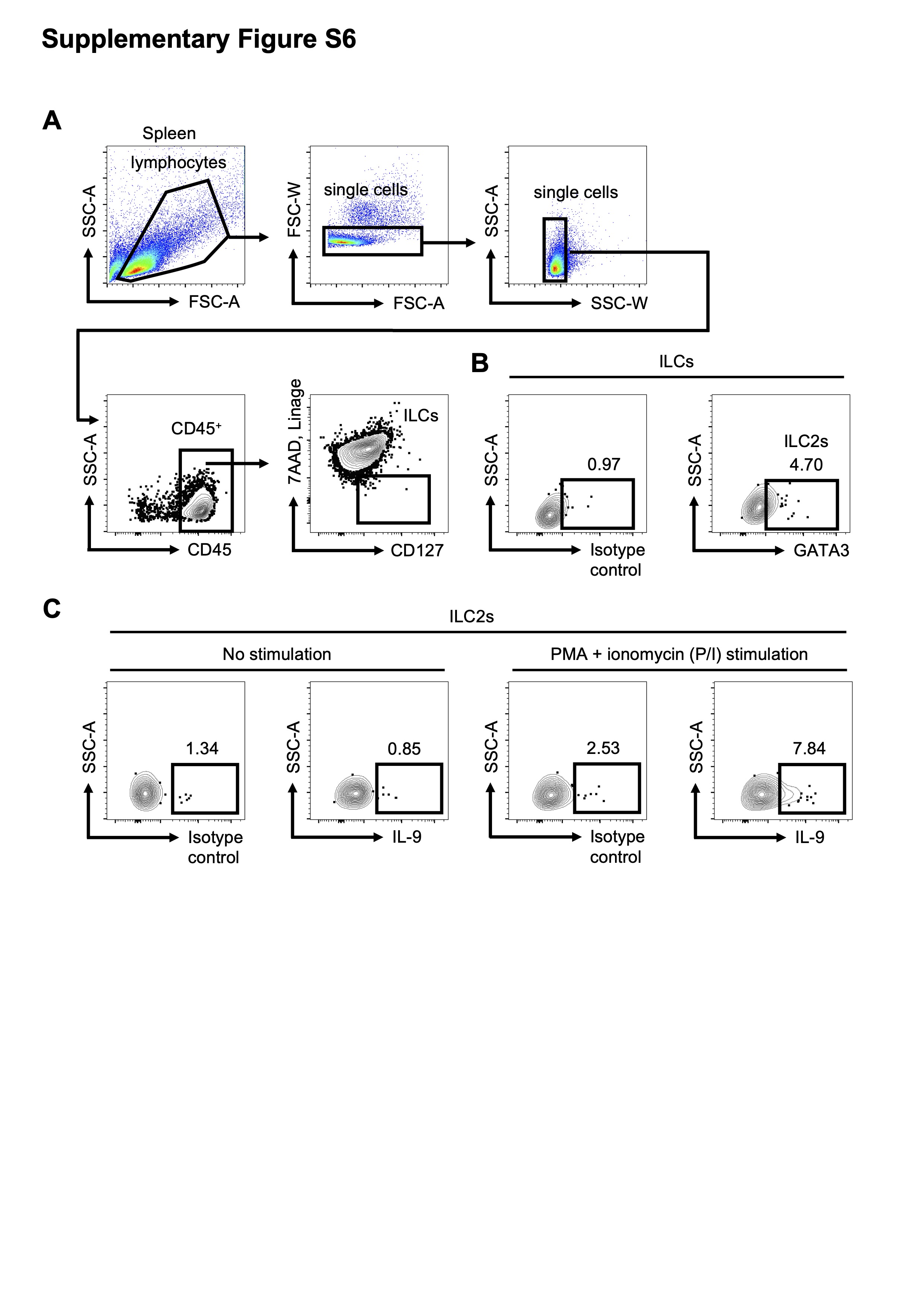

Supplement: Supplementary Figure 6 — Gating strategy for ILCs in FoxP3GFP/DTR mice (corresponding to Figure 4 ). (A) Gating strategy for ILCs. Lineage (Lin) markers include B220, CD3, CD11b, CD11c, Gr-1, NK1.1, TCRβ, TCRγδ, and TER119. (B) Representative FACS plots of GATA3+ ILCs (ILC2s) compared with isotype control. (C) Representative FACS plots of IL-9+ ILC2s compared with isotype control. [file Image6.jpeg]

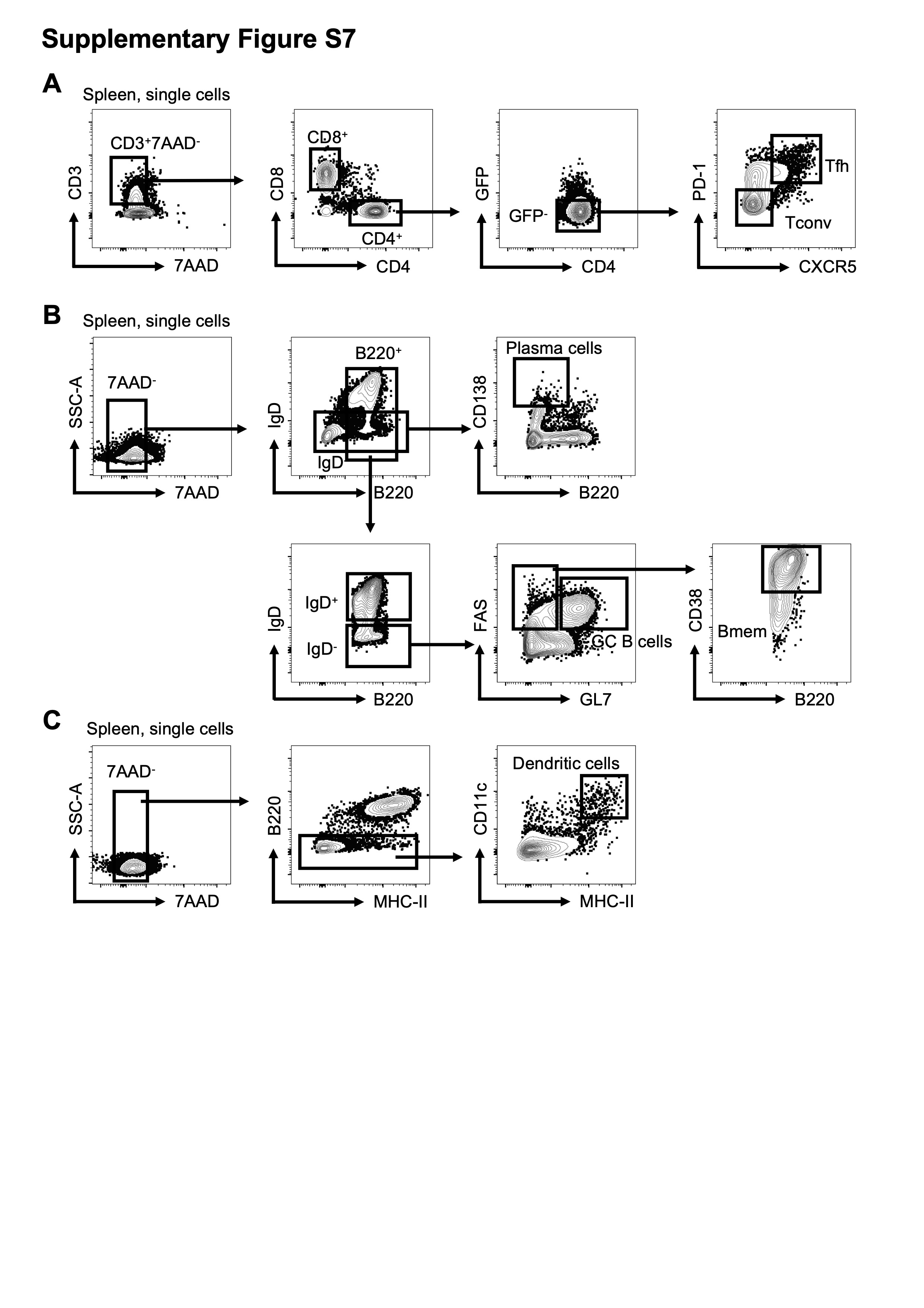

Supplement: Supplementary Figure 7 — Gating strategy for immune cell populations in FoxP3GFP/DTR mice (corresponding to Figure 4 ). (A) Gating strategy for T cell populations. (B) Gating strategy for B cell populations. (C) Gating strategy for dendritic cells. [file Image7.jpeg]

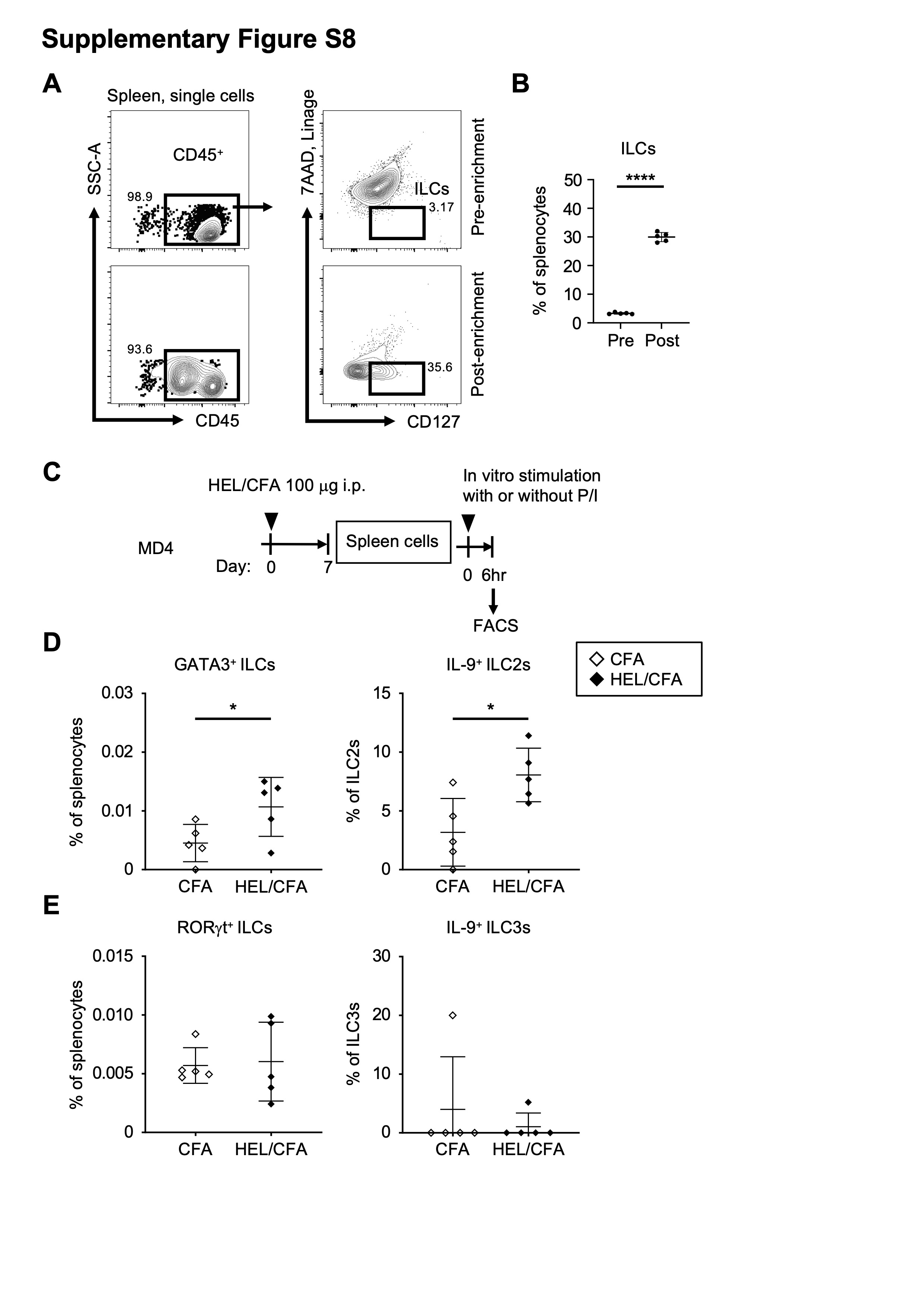

Supplement: Supplementary Figure 8 — Analyses of ILCs in wild-type and MD4 mice (corresponding to Figure 5 ). (A) Representative FACS plots of ILCs concerning the reanalysis of cells obtained from splenocytes using a mouse ILC2 enrichment kit. Lineage markers are represented as Lin; B220, CD3, CD11b, CD11c, Gr-1, NK1.1, TCRβ, TCRγδ, and TER119. (B) %ILCs in A. Each dot represents the average value of a group of mice (n = 3-4). ****p < 0.0001. Data represent the mean ± SD of 4-5 groups. (C) Experimental protocol for immunization of MD4 mice. 100 μg HEL/CFA was administered intraperitoneally. After the immunization, spleen cells were cultured with or without P/I for 6 hr. (D) Percentages of GATA3+ ILCs (ILC2s) in splenocytes, *p = 0.0490. Percentages of IL-9+ ILC2s in ILC2s, *p = 0.0317. (E) Percentages of RORgt ILCs (ILC3s) in splenocytes and IL-9+ ILC3s in ILCs. Data represent the mean ± SD of 5 mice per group. Statistical significance was analyzed using the unpaired t-test. Similar results were obtained in two independent experiments. [file Image8.jpeg]

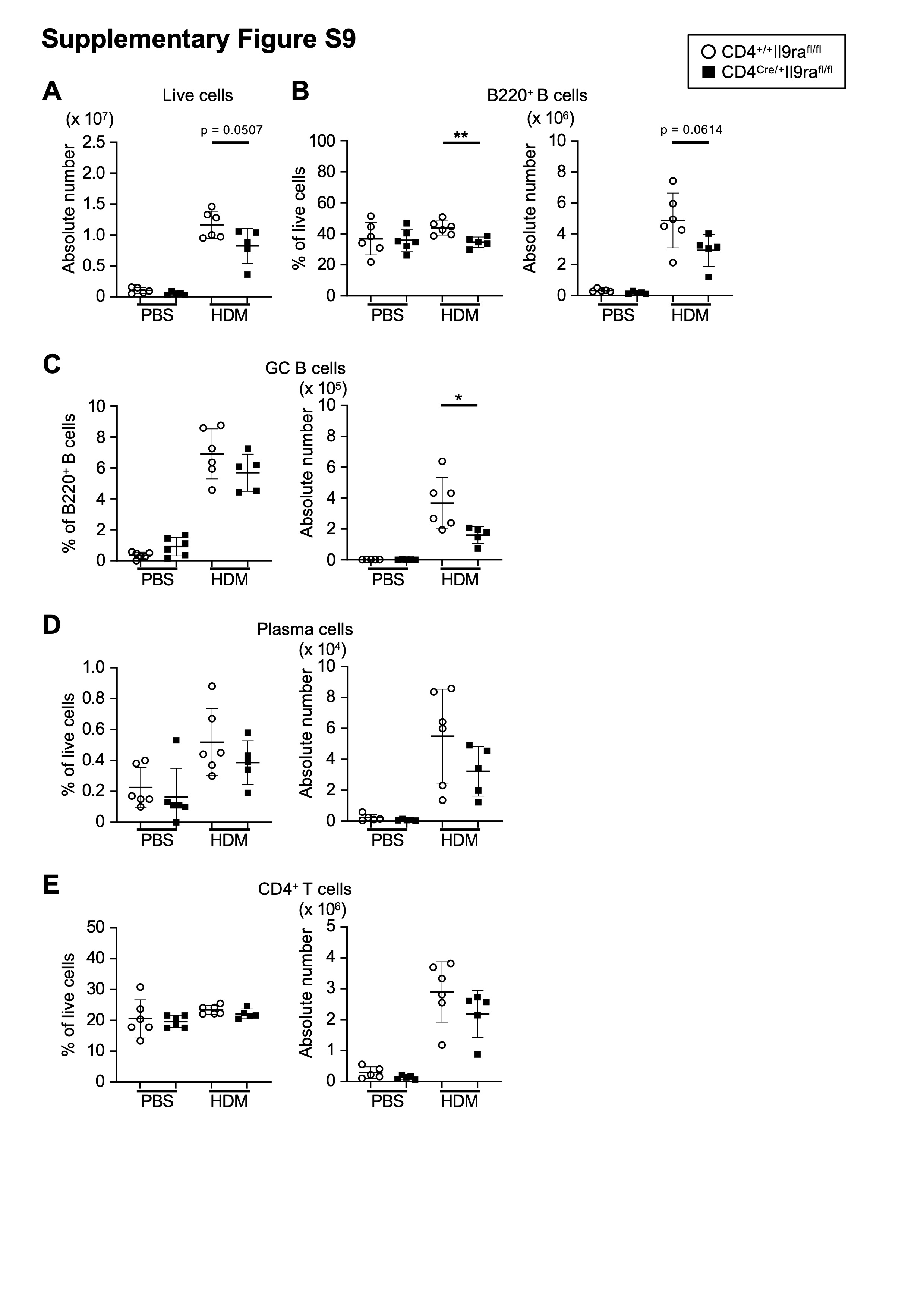

Supplement: Supplementary Figure 9 — Analysis of immune cells in mediastinal lymph nodes in HDM-induced allergic asthma models (corresponding to Figure 6 ). (A) Numbers of live cells in mediastinal lymph nodes, p = 0.0507. (B–E) Percentages and numbers of cell populations in mediastinal lymph nodes ( Supplementary Figures 3A, B ). (B) B220+ B cells. Percentages, **p = 0.0049; cell numbers, p = 0.0614. (C) GC B cells. Cell numbers, *p = 0.0264. (D) Plasma cells. (E) CD4+ T cells. Data represent the mean ± SD of 5-6 mice per group. Statistical significance was analyzed using the unpaired t-test (A–E). All data are from CD4+/+Il9rafl/fl (open circle) and CD4Cre/+Il9rafl/fl mice (filled square). Similar results were obtained in three independent experiments. [file Image9.jpeg]
